# A20 Facilitates Oxaliplatin Sensitivity in Colorectal Cancer Through Monoubiquitylation of IKK‐β

**DOI:** 10.1002/advs.202514486

**Published:** 2025-12-17

**Authors:** Fan Luo, Ting Yang, Jiaxin Cao, Qun Chen, Zhenhai Lu, Feiteng Lu, Chaozhuo Lin, Zengfei Xia, Yuanzhong Yang, Peng Li, Wenjuan Ma, Min Luo, Rongxin Zhang

**Affiliations:** ^1^ Department of Intensive Care Unit State Key Laboratory of Oncology in South China Guangdong Key Laboratory of Nasopharyngeal Carcinoma Diagnosis and Therapy Guangdong Provincial Clinical Research Center for Cancer Sun Yat‐sen University Cancer Center Guangzhou 510060 P. R. China; ^2^ Department of Clinical Research State Key Laboratory of Oncology in South China Guangdong Key Laboratory of Nasopharyngeal Carcinoma Diagnosis and Therapy Guangdong Provincial Clinical Research Center for Cancer Sun Yat‐sen University Cancer Center Guangzhou 510060 P. R. China; ^3^ Department of Anesthesiology State Key Laboratory of Oncology in South China Guangdong Key Laboratory of Nasopharyngeal Carcinoma Diagnosis and Therapy Guangdong Provincial Clinical Research Center for Cancer Sun Yat‐sen University Cancer Center Guangzhou 510060 P. R. China; ^4^ Department of Medical Oncology Fujian Medical University Union Hospital Fuzhou China; ^5^ Department of Colorectal Surgery State Key Laboratory of Oncology in South China Guangdong Key Laboratory of Nasopharyngeal Carcinoma Diagnosis and Therapy Guangdong Provincial Clinical Research Center for Cancer Sun Yat‐sen University Cancer Center Guangzhou 510060 P. R. China; ^6^ Department of Experimental Research State Key Laboratory of Oncology in South China Guangdong Key Laboratory of Nasopharyngeal Carcinoma Diagnosis and Therapy Guangdong Provincial Clinical Research Center for Cancer Sun Yat‐sen University Cancer Center Guangzhou 510060 P. R. China; ^7^ Department of Pathology State Key Laboratory of Oncology in South China Guangdong Key Laboratory of Nasopharyngeal Carcinoma Diagnosis and Therapy Guangdong Provincial Clinical Research Center for Cancer Sun Yat‐sen University Cancer Center Guangzhou 510060 P. R. China; ^8^ Department of Clinical Laboratory State Key Laboratory of Oncology in South China Guangdong Key Laboratory of Nasopharyngeal Carcinoma Diagnosis and Therapy Guangdong Provincial Clinical Research Center for Cancer Sun Yat‐sen University Cancer Center Guangzhou 510060 P. R. China

**Keywords:** A20, chemoresistance, colorectal cancer, IKK‐β, oxaliplatin

## Abstract

The ubiquitin‐editing enzyme A20 is essential for maintaining inflammatory homeostasis, yet its role in chemoresistance remains unclear. To investigate how A20 regulates oxaliplatin resistance in colorectal cancer (CRC), with a focus on A20‐mediated IKK‐β monoubiquitylation at K163. A prospective, randomized Phase III study is conducted in patients with locally advanced CRC who received either oxaliplatin+capecitabine neoadjuvant chemoradiotherapy (oxaliplatin‐NACRT) or capecitabine‐only neoadjuvant chemoradiotherapy (non‐oxaliplatin‐NACRT). Preoperative biopsy and matched surgical specimens are evaluated by immunohistochemistry to determine A20's association with oxaliplatin response. In oxaliplatin‐NACRT group, patients achieving pathological complete response (pCR) displayed lower A20 expression than non‐pCR patients, whereas no difference is observed in non‐oxaliplatin‐NACRT group. Two additional independent cohorts confirmed A20 downregulation in human CRC tissues, and reduced A20 expression correlated with poorer survival and oxaliplatin resistance. Mechanistically, A20 monoubiquitylates IKK‐β via its fourth zinc‐finger domain, promoting IKK‐β degradation and suppressing NF‐κB nuclear translocation. A20 knockdown or expression of the IKK‐β K163R mutant induced oxaliplatin resistance in mouse xenografts. Clinically, A20 expression are negatively associated with IKK‐β and its downstream targets. A20 depletion promotes oxaliplatin resistance in CRC by stabilizing IKK‐β. the results uncover an essential role of the A20‐IKK‐β axis in oxaliplatin resistance of CRC.

## Graphical abstract

This study demonstrates that the expression of A20, a ubiquitin‐editing enzyme, is positively correlated with oxaliplatin sensitivity in colorectal cancer (CRC). Mechanistically, A20 enhances oxaliplatin sensitivity by inhibiting NF‐κB nuclear translocation through monoubiquitination of IKK‐β. The study identifies the A20–IKK‐β axis as a potential therapeutic target for overcoming oxaliplatin resistance in CRC.

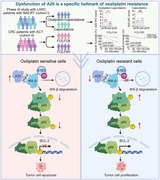

## 1 Introduction

Colorectal cancer (CRC) is a major cause of cancer‐related mortality worldwide.^[^ ^1^ ^]^ Chemotherapy remains a cornerstone of CRC management, and oxaliplatin—a third‐generation platinum compound—is routinely used as both a first‐line and adjuvant chemotherapeutic agent.^[^ ^2^ ^]^ Although initially effective, ≈50% of stage II and III CRC patients eventually develop resistance to oxaliplatin‐based adjuvant therapy.^[^ ^3^ ^]^ This highlights the urgent need to elucidate the mechanisms underlying oxaliplatin resistance and to develop strategies that improve therapeutic outcomes.

Multiple biological processes, including apoptosis, DNA repair, hypoxia, cellular metabolism, and epigenetic modification, contribute to oxaliplatin resistance.^[^ ^4^, ^5^, ^6^, ^7^ ^]^ Accumulating evidence also implicates the ubiquitin‐proteasome system (UPS) in tumor progression and chemoresistance.^[^ ^8^ ^]^ For instance, the thiophenyl compound P22077, a ubiquitin‐specific protease inhibitor, has been shown to enhance tumor cell sensitivity to doxorubicin (Dox) sensitivity in hepatocellular carcinoma (HCC) and pancreatic ductal adenocarcinoma (PDAC).^[^ ^9^, ^10^ ^]^ Thus, targeting the UPS represents a promising strategy to overcome therapeutic resistance. However, its involvement in oxaliplatin resistance in CRC remains insufficiently explored.

A20, also known as tumor necrosis factor alpha‐induced protein 3 (TNFAIP3), is a deubiquitinating enzyme and key regulator of immune and inflammatory signaling, functioning as both an anti‐apoptotic molecule and an inhibitor of nuclear factor‐kappa B (NF‐κB).^[^ ^11^, ^12^, ^13^, ^14^, ^15^ ^]^ Structurally, A20 contains C‐terminal zinc finger motifs with E3 ligase activity and an N‐terminal ovarian tumor (OTU) domain that mediates deubiquitination.^[^ ^16^, ^17^ ^]^ Although its role in inflammation is well established, A20's function in cancer remains controversial. Some studies report oncogenic roles for A20 across several tumor types,^[^ ^18^, ^19^, ^20^, ^21^, ^22^ ^]^ whereas others demonstrate tumor‐suppressive functions, particularly in B‐cell lymphoma^[^ ^23^, ^24^, ^25^ ^]^ and CRC.^[^ ^26^ ^]^ Evidence regarding its role in chemotherapy resistance is limited. One study found that A20 overexpression promotes resistance to etoposide and radiotherapy by inhibiting the E3 ligase RNF168.^[^ ^27^ ^]^ However, whether and how A20 contributes to oxaliplatin resistance has not been fully elucidated.

In this study, we identify A20 inactivation as a previously unrecognized driver of oxaliplatin resistance in CRC. Using immunohistochemistry (IHC) data from a Phase III clinical trial comparing oxaliplatin‐based and non‐oxaliplatin neoadjuvant chemoradiotherapy (NACRT), together with a retrospective cohort of 100 patients treated with FOLFOX or FOLFIRI adjuvant chemotherapy, we demonstrate that reduced A20 expression is associated with poor therapeutic response and unfavorable clinical outcomes. Functional analyses further show that loss of A20 induces oxaliplatin resistance in CRC cell lines and mouse xenografts. Mechanistically, A20 interacts with IKK‐β and suppresses NF‐κB/BCL‐2 signaling by promoting monoubiquitylation of IKK‐β, a process dependent on the A20 zinc finger 4 (ZnF4) domain and abolished by the K163R mutation in IKK‐β. Clinically, A20 expression negatively correlates with IKK‐β, p‐NF‐κB‐p65, and BCL‐2, and positively correlates with oxaliplatin response and prognosis. Collectively, our findings highlight A20 as a potential therapeutic target for reversing oxaliplatin resistance in CRC.

## 2 Results

### 2.1 A20 Dysfunction is a Hallmark of Oxaliplatin Resistance

The study cohorts were obtained from the SONCAR Phase III clinical trial (NCT02031939), which enrolled patients with locally advanced rectal cancer (LARC) patients (cohort I).

**Figure 1:**
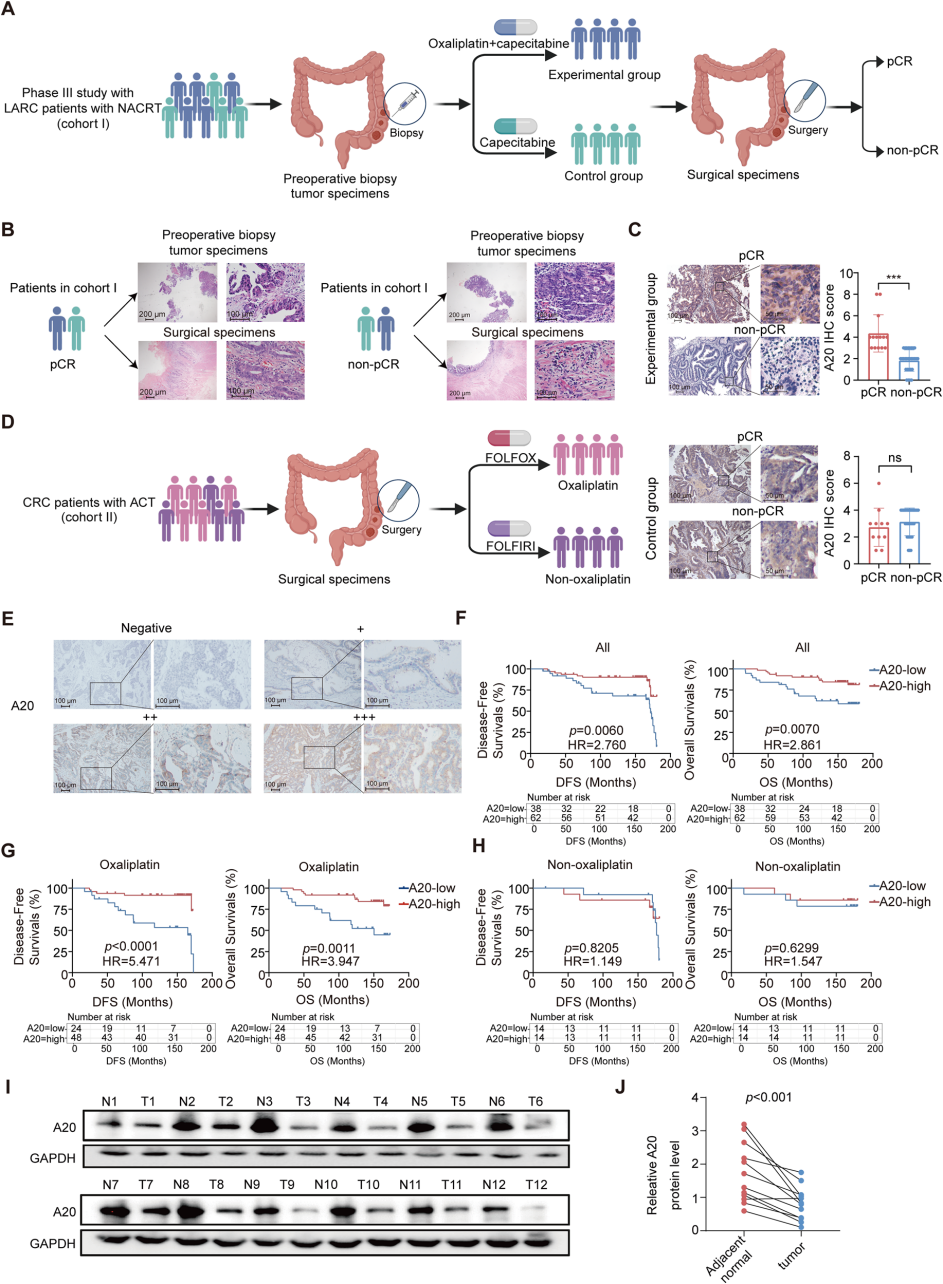
A20 dysfunction is associated with poor oxaliplatin response in CRC patients.A) Schematic diagram of the prospective randomized controlled Phase III study in which patients with LARC received neoadjuvant chemoradiotherapy consisting of oxaliplatin plus capecitabine (experimental group) or capecitabine alone (control group).B). Representative hematoxylin‐eosin (H&E) staining images from 85 LARC patients in cohort I. Patients were classified as pCR or non‐pCR based on pathological tumor regression grade.C). Representative images and quantification of A20 IHC staining in LARC patients treated with oxaliplatin plus capecitabine (experimental group: pCR, n = 14; non‐pCR, n = 29) or capecitabine alone (control group: pCR, n = 11; non‐pCR, n = 31) in cohort I.D). Schematic diagram depicting the study design for CRC patients in cohort II who underwent radical surgery followed by FOLFOX (oxaliplatin‐based) or FOLFIRI (non‐oxaliplatin) adjuvant chemotherapy.E). Representative IHC staining images of A20 expression in CRC patients from cohort II who received radical surgery followed by FOLFOX or FOLFIRI therapy. Staining intensity was scored as follows: ‐ (negative), + (weak), ++ (moderate), +++ (strong).F). Kaplan‒Meier curves of DFS and OS in cohort II patients treated with FOLFOX or FOLFIRI, stratified by A20 protein expression (Low A20, n =38; High A20, n = 62).G). Kaplan‐Meier analysis of DFS and OS in cohort II patients treated with FOLFOX adjuvant chemotherapy, stratified by A20 protein expression (Low A20, n =24; High A20, n = 48).H). Kaplan‐Meier analysis of DFS and OS in cohort II patients treated with FOLFIRI adjuvant chemotherapy, stratified by A20 protein expression (Low A20, n =14; High A20, n = 14).I‐J). Western blot analysis (I) and quantification (J) of A20 expression in 12 paired CRC tumor tissues (T) and adjacent normal tissues (N).

In this trial, patients in the experimental arm received induction chemotherapy with oxaliplatin plus capecitabine prior to radiotherapy, whereas patients in the control arm received capecitabine alone (**Figure** **1**A). Paired preoperative biopsy and postoperative surgical specimens were collected from 85 patients (43 experimental, 42 control) to investigate the association between A20 expression and oxaliplatin response. Baseline characteristics for these patients are listed in Table S1 (Supporting Information). Among the 85 patients, 25 achieved pathological complete response (pCR; 14 in the experimental group and 11 in the control group), and 60 were non‐pCR (29 in the experimental group and 31 in the control group) based on tumor regression grade (Figure 1B). A20 expression was significantly reduced in non‐pCR patients compared with pCR patients within the experimental group, whereas no difference was detected in the control group (Figure 1C). These findings indicate that reduced A20 expression might be correlated to oxaliplatin resistance.

To assess the consistency, we next performed IHC analysis of A20 expression in an independent cohort of 100 CRC patients who underwent radical surgery and received adjuvant chemotherapy (cohort II) (Figure 1D,E). Of these patients, 72 received FOLFOX (folinic acid, fluorouracil, and oxaliplatin), and 28 received FOLFIRI (folinic acid, fluorouracil, and irinotecan). Kaplan–Meier survival analysis revealed that low A20 expression was associated with significantly shorter disease‐free survival (DFS) and overall survival (OS) compared with high A20 expression (*p* = 0.0060 for DFS, HR = 2.760 (95% CI, 1.327‐5.739); *p* = 0.0070 for OS, HR = 2.861 (95% CI, 1.254‐6.530); Figure 1F). Subgroup analysis showed that A20 expression highly predicted DFS (*p* < 0.0001, HR = 5.471 (95% CI, 2.036‐14.70) and OS (*p* = 0.0011, HR = 3.947 (95% CI, 1.480‐10.53)) in patients treated with FOLFOX (Figure 1G), whereas A20 expression did not influence DFS (*p* = 0.8205, HR = 1.149 (95% CI, 0.3433‐3.847)) or OS (*p* = 0.6299, HR = 1.547 (95% CI, 0.2679‐8.931)) in patients treated with FOLFIRI (Figure 1H). Univariate and multivariate Cox regression analyses further identified A20 as an independent prognostic factor (Tables S2–S4, Supporting Information).

Consistent with these observations, Western blot analysis of 12 paired CRC tumor and adjacent normal tissues (cohort III) showed markedly lower A20 protein levels in tumor samples (Figure 1I,J). Together, these clinical and molecular findings collectively suggest that low A20 expression is closely associated with oxaliplatin resistance and unfavorable prognosis in CRC.

### 2.2 Loss of A20 Confers Oxaliplatin Resistance to CRC In Vitro and In Vivo

**Figure 2:**
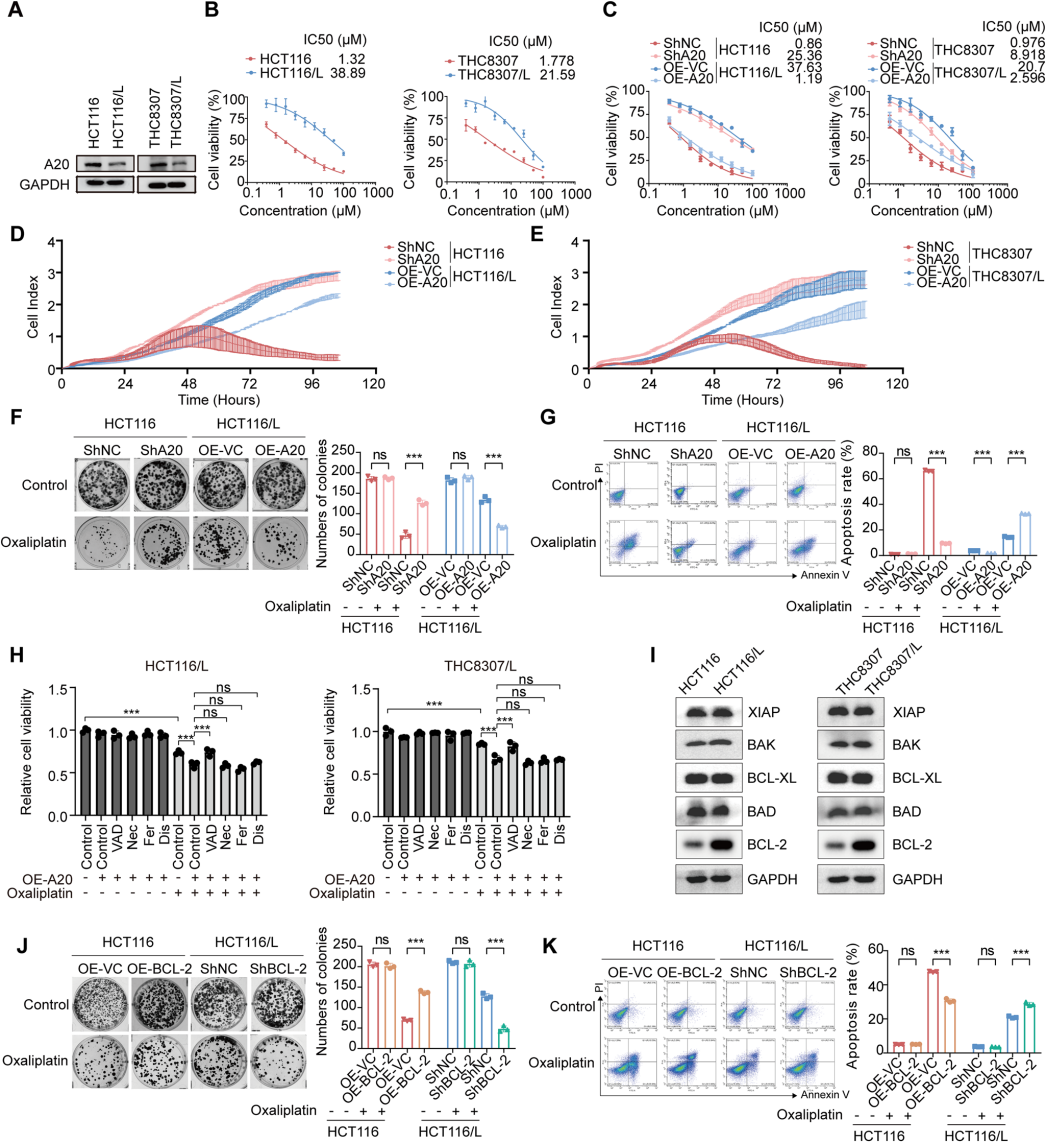
Loss of A20 confers oxaliplatin resistance through regulation of BCL‐2.A). Western blot analysis of A20 in HCT116, HCT116/L, THC8307, and THC8307/L cells. A20 protein expression is shown in oxaliplatin‐sensitive CRC cell lines (HCT116, THC8307) and their corresponding oxaliplatin‐resistant derivatives (HCT116/L, THC8307/L).B). IC_50_ values for oxaliplatin in HCT116, HCT116/L, THC8307, and THC8307/L cells, demonstrating significantly increased resistance in HCT116/L and THC8307/L cells.C). IC_50_ values for oxaliplatin in HCT116‐shNC, HCT116‐shA20, HCT116/L‐OE‐VC, HCT116/L‐OE‐A20, THC8307‐shNC, THC8307‐shA20, THC8307/L‐OE‐VC, and THC8307/L‐OE‐A20 cells.D‐E). Cell growth curves of HCT116‐shNC, HCT116‐shA20, HCT116/L‐OE‐VC, HCT116/L‐OE‐A20, THC8307‐shNC, THC8307‐shA20, THC8307/L‐OE‐VC, and THC8307/L‐OE‐A20 cells over 100 h. Cells were seeded in E‐plates (1000 cells per well), and the cell index was measured every 15 min for 100 h (n = 2).F). Representative images and quantification of colony formation in HCT116‐shNC, HCT116‐shA20, HCT116/L‐OE‐VC, and HCT116/L‐OE‐A20 cells treated with control or oxaliplatin (n = 3).G). Representative images and quantification of annexin V/propidium iodide staining in HCT116‐shNC, HCT116‐shA20, HCT116/L‐OE‐VC, and HCT116/L‐OE‐A20 cells treated with control or oxaliplatin (n = 3).H). Relative cell viability of HCT116/L and THC8307/L cells overexpressing A20, treated with control, oxaliplatin, the apoptosis inhibitor Z‐VAD‐FMK (VAD), the necrosis inhibitor necrostatin (Nec), the ferroptosis inhibitor ferrostatin‐1 (Fer), or the pyroptosis inhibitor disulfiram (Dis) (n = 3).I). Western blot analysis of XIAP, BAK, BCL‐XL, BAD, and BCL‐2 expression in HCT116, HCT116/L, THC8307, and THC8307/L cells.J). Representative images and quantification of colony formation in HCT116‐OE‐VC, HCT116‐OE‐BCL‐2, HCT116/L‐shNC, and HCT116/L‐shBCL‐2 cells treated with control or oxaliplatin (n = 3).K). Representative images and quantification of annexin V/propidium iodide staining in HCT116‐OE‐VC, HCT116‐OE‐BCL‐2, HCT116/L‐shNC, and HCT116/L‐shBCL‐2 cells treated with control or oxaliplatin (n = 3).Data are presented as mean ± SD; ns, no significance, *p < 0.05, **p < 0.01, ***p < 0.001.

To determine the role of A20 in oxaliplatin sensitivity, we first examined its expression in two CRC cell lines (HCT116, THC8307) and their oxaliplatin‐resistant derivatives (HCT116/L, THC8307/L). A20 expression was markedly downregulated in the oxaliplatin‐resistant cell lines (HCT116/L and THC8307/L) (**Figure** **2**A). We then evaluated the oxaliplatin sensitivity of these cells. Consistent with their resistant phenotype, HCT116/L and THC8307/L cells exhibited 20‐40‐fold higher IC_50_ values for oxaliplatin compared with their parental counterparts (Figure 2B).

To further validate the role of A20 in oxaliplatin sensitivity, we generated stable A20 knockdown cells (shA20) from oxaliplatin‐sensitive CRC cell lines (HCT116 and THC8307), and A20‐overexpressing cells (OE‐A20) from their resistant counterparts (HCT116/L and THC8307/L) (Figure S1A, Supporting Information). Cytotoxicity assays performed over 72 h showed that A20 knockdown in HCT116 and THC8307 cells reduced oxaliplatin sensitivity, as indicated by higher IC_50_ values relative to vector controls (shNC) (Figure 2C). Conversely, A20 overexpression in HCT116/L and THC8307/L cells increased oxaliplatin sensitivity, reflected by lower IC_50_ values compared with the vector control (OE‐VC) (Figure 2C).

We next examined whether A20 influences CRC cell proliferation under oxaliplatin treatment using real‐time cell analysis (RTCA). Consistent with the cytotoxicity results, A20 knockdown attenuated oxaliplatin‐induced growth inhibition in the sensitive cell lines (HCT116 and THC8307), whereas A20 overexpression enhanced proliferation inhibition in the resistant cell lines (HCT116/L and THC8307/L) (Figure 2D,E). Colony formation assays further demonstrated that, upon oxaliplatin treatment, shA20 cells formed more colonies than shNC control cells in both HCT116 and THC8307 backgrounds. In contrast, A20 overexpression markedly decreased colony numbers in HCT116/L and THC8307/L cells compared with OE‐VC controls (Figures 2F; S1B, Supporting Information).

Since CRC recurrence is largely driven by chemotherapy resistance and dysregulated apoptosis,^[^ ^28^, ^29^ ^]^ we next examined whether A20 influences apoptotic responses in CRC. Annexin V/PI staining revealed that A20 knockdown in oxaliplatin‐sensitive cells reduced apoptosis, whereas A20 overexpression in resistant cells enhanced oxaliplatin‐induced apoptosis (Figures 2G; S1C, Supporting Information). These results highlight a critical role for A20 in modulating oxaliplatin sensitivity through apoptosis regulation.

To further determine the type of cell death associated with A20 upregulation, we treated CRC cells with oxaliplatin in the presence of specific cell death inhibitors. Notably, the apoptosis inhibitor Z‐VAD‐FMK reversed the decreased cell viability caused by A20 overexpression under oxaliplatin treatment, whereas of necroptosis (necrostatin‐1), ferroptosis (ferrostatin‐1), and pyroptosis (disulfiram) showed no such effect (Figure 2H). These findings suggest that A20 overexpression primarily sensitizes CRC cells to oxaliplatin through apoptosis induction.

We next investigated how A20 regulates apoptotic pathways at the molecular level following oxaliplatin exposure. Among BCL‐2 family proteins, only BCL‐2 expression was markedly elevated in resistant CRC cells compared with parental cells, while levels of XIAP, BAK, BCL‐XL, and BAD remained unchanged (Figure 2I). To assess whether BCL‐2 contributes to oxaliplatin resistance, we generated CRC cells with stable BCL‐2 overexpression or knockdown (Figure S2A, Supporting Information). CCK‐8 assays showed that BCL‐2 overexpression promoted cell proliferation under oxaliplatin treatment, whereas BCL‐2 knockdown significantly suppressed cell growth (Figure S2B–E, Supporting Information). Plate cloning and Annexin V/PI assays confirmed that BCL‐2 overexpression reduced oxaliplatin sensitivity, while BCL‐2 knockdown increased apoptosis and sensitized cells to oxaliplatin (Figures 2J,K; S2F,G, Supporting Information). Furthermore, the BCL‐2 inhibitor APG‐2575 markedly inhibited proliferation and increased apoptosis in oxaliplatin‐resistant CRC cells exposed to oxaliplatin (Figure S2H,I, Supporting Information). Together, these results indicate that A20 enhances oxaliplatin‐induced apoptosis at least in part through the modulation of BCL‐2.

**Figure 3:**
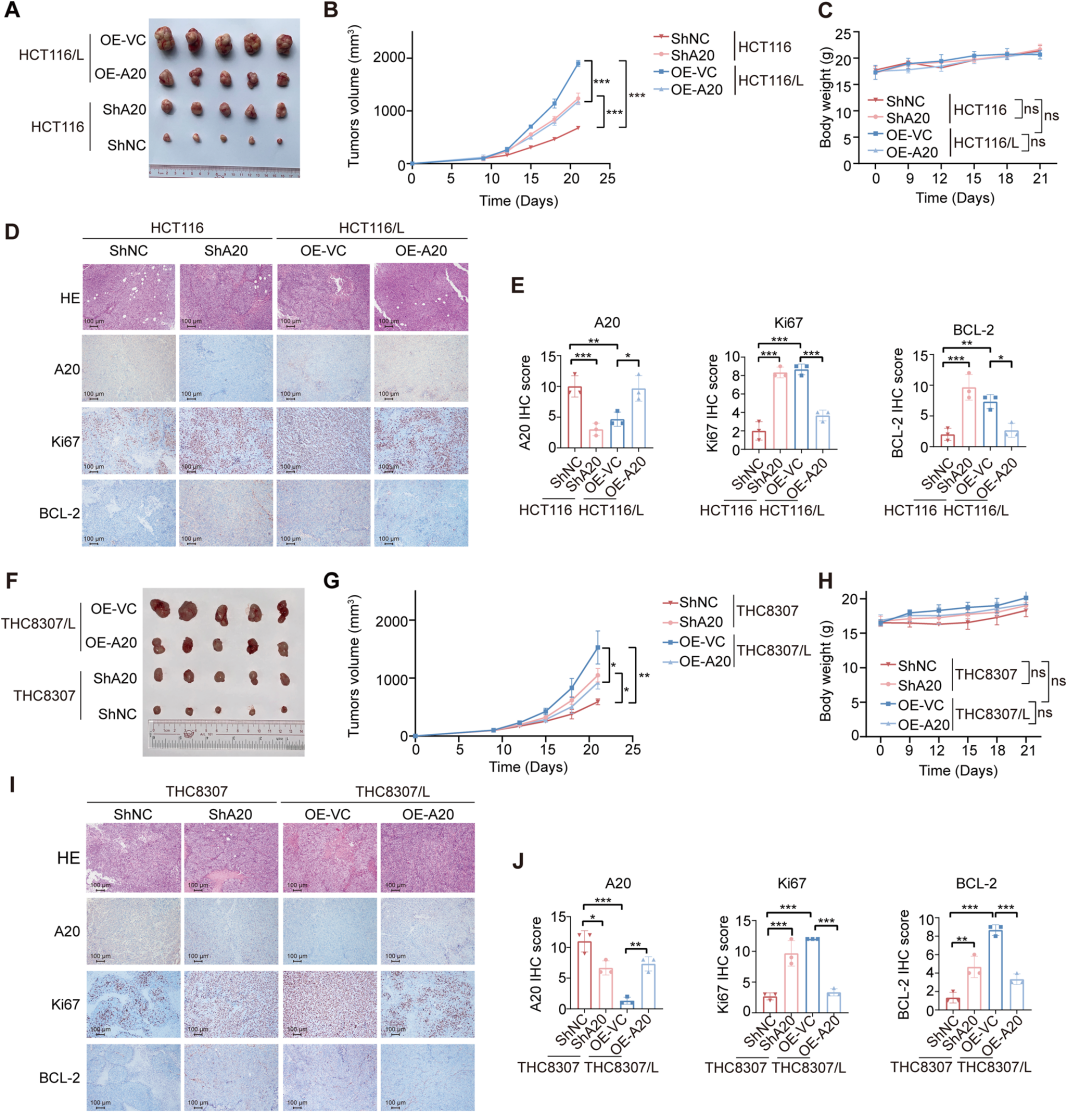
A20 mediates oxaliplatin sensitivity via regulation of BCL‐*2* in vivo.A). Tumor images from mice treated with oxaliplatin in the HCT116/L‐OE‐VC, HCT116/L‐OE‐A20, HCT116‐shA20, and HCT116‐shNC groups in tumor‐bearing BALB/c nude mice (n = 5).B‐C). Tumor outgrowth (B) and body weight (C) of mice treated with oxaliplatin in the HCT116‐shNC, HCT116‐shA20, HCT116/L‐OE‐VC, and HCT116/L‐OE‐A20 groups in tumor‐bearing BALB/c nude mice (n = 5).D. Representative images of H&E staining and immunohistochemical staining for A20, Ki67, and BCL‐2 in tumor tissues from oxaliplatin‐treated mice in the HCT116‐shNC, HCT116‐shA20, HCT116/L‐OE‐VC, and HCT116/L‐OE‐A20 groups.E). Quantification of immunohistochemical staining for A20, Ki67, and BCL‐2 in tumor tissues from oxaliplatin‐treated mice in the HCT116‐shNC, HCT116‐shA20, HCT116/L‐OE‐VC, and HCT116/L‐OE‐A20 groups (n = 3).F). Tumor images from mice treated with oxaliplatin in the THC8307/L‐OE‐VC, THC8307/L‐OE‐A20, THC8307‐shA20, and THC8307‐shNC groups in tumor‐bearing BALB/c nude mice (n = 5).G‐H). Tumor outgrowth (G) and body weight (H) of mice treated with oxaliplatin in the THC8307‐shNC, THC8307‐shA20, THC8307/L‐OE‐VC, and THC8307/L‐OE‐A20 groups in tumor‐bearing BALB/c nude mice (n = 5).I). Representative images of H&E staining and immunohistochemical staining for A20, Ki67, and BCL‐2 in tumor tissues from oxaliplatin‐treated mice in the THC8307‐shNC, THC8307‐shA20, THC8307/L‐OE‐VC, and THC8307/L‐OE‐A20 groups.J). Quantification of immunohistochemical staining for A20, Ki67, and BCL‐2 in tumor tissues from oxaliplatin‐treated mice in the THC8307‐shNC, THC8307‐shA20, THC8307/L‐OE‐VC, and THC8307/L‐OE‐A20 groups (n = 3).Data are presented as mean ± SD; ns, no significance, ^*^ *p *< 0.05, ^**^ *p* < 0.01, ^***^ *p* < 0.001.

To further assess the role of A20 in oxaliplatin sensitivity in vivo, we established a xenograft mouse model. BALB/c nude mice were inoculated with HCT116/THC8307‐shNC, HCT116/THC8307‐shA20, HCT116/L/THC8307/L‐OE‐VC, or HCT116/LTHC8307/L‐OE‐A20 cells and treated with 10 mg kg^−1^ oxaliplatin once weekly for three weeks. Tumor volumes were measured every three days. As expected, xenografts derived from resistant cells (HCT116/ THC8307/L‐OE‐VC) exhibited significantly larger tumor volumes than those derived from parental shNC controls, confirming their resistant phenotype. A20 overexpression markedly suppressed tumor growth in resistant xenografts, whereas A20 knockdown enhanced tumor growth in parental xenografts (**Figure** **3**A–C, F–H). Immunohistochemical staining further validated the efficiency of A20 knockdown and overexpression in vivo (Figure 3D, E, I,J).

Furthermore, Ki67 levels were elevated in oxaliplatin‐resistant CRC xenografts compared with parental xenografts. Ki67 expression was further increased in parental tumors following A20 knockdown and reduced in resistant tumors upon A20 overexpression (Figure 3D, E, I, J). Consistently, IHC staining showed that BCL‐2 expression was upregulated in xenografts derived from A20‐deficient cells and downregulated in xenografts derived from A20‐overexpressing resistant cells (Figure 3D, E, I, J). Together, these findings indicate that A20 regulates oxaliplatin sensitivity in vivo at least in part through modulation of BCL‐2.

### 2.3 A20 Reduces BCL‐2 Expression in an IKK‐β‐Dependent Manner

**Figure 4:**
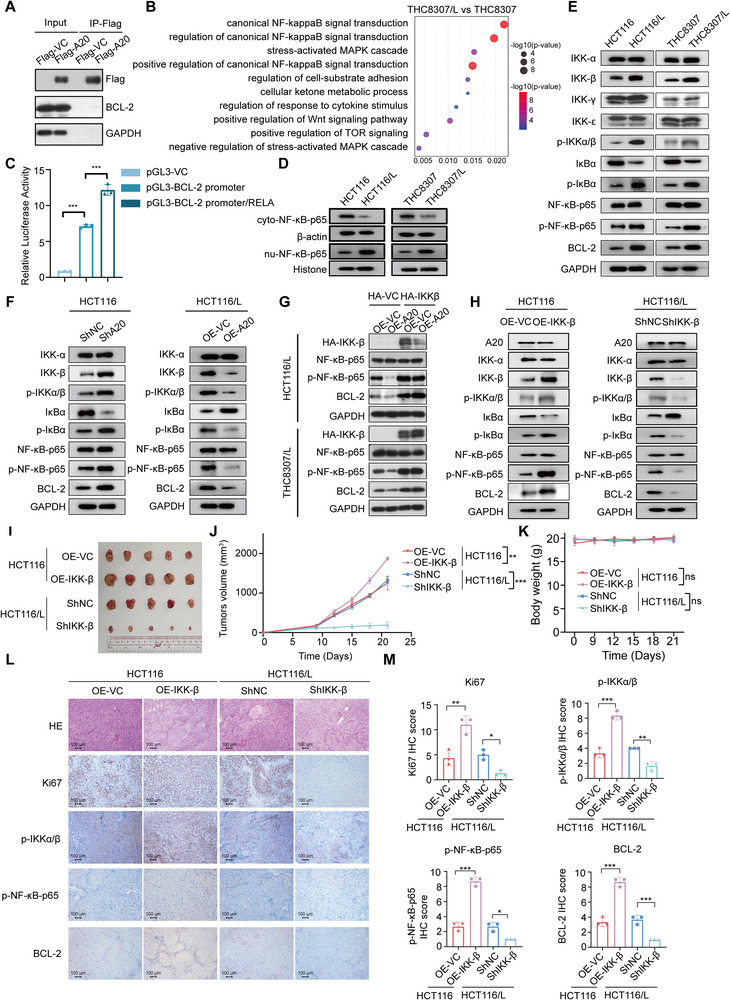
A20 regulates oxaliplatin resistance via the IKK‐β/NF‐κB/BCL‐2 axis.A). Immunoprecipitation followed by western blot analysis of the interaction between Flag‐tagged‐vector (Flag‐VC) and Flag‐tagged‐A20 (Flag‐A20) with BCL‐2 in HEK293T cells.B). GO pathway enrichment analysis of differential gene expression between THC8307 and THC8307/L cells.C). Luciferase activity (normalized to Renilla luciferase) in HEK293T cells transiently transfected for 48 h with pGL3‐Basic, pGL3‐Basic containing the BCL‐2 promoter, or pGL3‐Basic containing the BCL‐2 promoter plus pCMV‐RELA (n = 3).D). Western blot analysis of NF‐κB‐p65 in cytoplasmic (cyto) and nuclear (nu) fractions of HCT116, HCT116/L, THC8307, and THC8307/L cells.E). Western blot analysis of IKK‐α, IKK‐β, IKK‐γ, IKK‐ε, p‐IKKα/β, IκBα, p‐IκBα, NF‐κB‐p65, p‐NF‐κB‐p65, and BCL‐2 expression in HCT116, HCT116/L, THC8307, and THC8307/L cells.F). Western blot analysis of IKK‐α, IKK‐β, p‐IKKα/β, IκBα, p‐IκBα, NF‐κB‐p65, p‐NF‐κB‐p65, and BCL‐2 expression in HCT116 cells with stable A20 knockdown and HCT116/L cells with A20 overexpression.G). Western blot analysis of HA‐tagged‐IKK‐β, NF‐κB‐p65, p‐NF‐κB‐p65, and BCL‐2 expression in stable A20 overexpression HCT116/L and THC8307/L cells with or without IKK‐β overexpression.H). Western blot analysis of A20, IKK‐α, IKK‐β, p‐IKKα/β, IκBα, p‐IκBα, NF‐κB‐p65, p‐NF‐κB‐p65, and BCL‐2 expression in HCT116‐OE‐VC, HCT116‐OE‐IKK‐β, HCT116/L‐shNC, and HCT116/L‐shIKK‐β cells.I). Tumor images from BALB/c nude mice treated with oxaliplatin in the HCT116‐OE‐VC, HCT116‐OE‐IKK‐β, HCT116/L‐shNC, and HCT116/L‐shIKK‐β groups (n = 5).J‐K). Tumor outgrowth (J) and body weight (K) of mice treated with oxaliplatin in the HCT116‐OE‐VC, HCT116‐OE‐IKK‐β, HCT116/L‐shNC, and HCT116/L‐shIKK‐β groups (n = 5).L). Representative images of H&E and immunohistochemical staining for Ki67, p‐IKKα/β, p‐NF‐κB‐p65, and BCL‐2 in tumor tissues from oxaliplatin‐treated mice in the HCT116‐OE‐VC, HCT116‐OE‐IKK‐β, HCT116/L‐shNC, and HCT116/L‐shIKK‐β groups.M). Quantification of Ki67, p‐IKKα/β, p‐NF‐κB‐p65, and BCL‐2 immunohistochemical staining in tumor tissues from oxaliplatin‐treated mice in the HCT116‐OE‐VC, HCT116‐OE‐IKK‐β, HCT116/L‐shNC, and HCT116/L‐shIKK‐β groups (n = 3).Data are presented as mean ± SD; ns, no significance, ^*^ *p *< 0.05, ^**^ *p* < 0.01, ^***^ *p* < 0.001.

To investigate the mechanism through which A20 regulates BCL‐2 expression, we first examined whether A20 directly interacts with BCL‐2. Co‐immunoprecipitation analyses showed no direct binding between the two proteins (**Figure** **4**A). RNA‐seq was then performed in oxaliplatin‐sensitive (THC8307) and oxaliplatin‐resistant (THC8307/L) cell lines. Gene Ontology (GO) analysis revealed significant enrichment of pathways related to canonical NF‐kappaB signaling, including “canonical NF‐kappaB signal transduction” and “positive regulation of canonical NF‐kappaB signal transduction” in resistant cells (Figure 4B). Previous studies have shown that RELA (NF‐κB‐p65), a key NF‐kB family member, plays essential roles in cell survival, apoptosis, differentiation, and stress responses,^[^ ^30^, ^31^ ^]^ and that nuclear translocation of NF‐κB‐p65 is required for NF‐κB pathway activation and transcriptional induction of BCL‐2.^[^ ^32^, ^33^, ^34^ ^]^

To determine whether A20 modulates BCL‐2 expression through NF‐κB‐p65, we performed luciferase reporter assays, which confirmed that NF‐κB‐p65 binds to the BCL‐2 promoter to enhance its transcription (Figure 4C). Consistent with this, NF‐κB‐p65 nuclear translocation was markedly increased in oxaliplatin‐resistant cells and in A20‐knockdown cells, whereas A20 overexpression significantly reduced NF‐kB‐p65 translocation (Figures 4D; S3A, Supporting Information). Together, these findings suggest that A20 suppresses BCL‐2 expression by inhibiting NF‐κB‐p65 activation and its binding to the BCL‐2 promoter.

These findings prompted us to further investigate how A20 modulates NF‐κB‐p65 activity. Analysis of upstream components of the NF‐κB pathway revealed that IKK‐β, together with p‐IKK‐α/β, p‐IκBα, p‐NF‐κB‐p65, and BCL‐2, was upregulated in oxaliplatin‐resistant cells, whereas the levels of other related molecules (IKK‐α, IKK‐γ, IKK‐ε) remained unchanged (Figure 4E). Manipulating A20 expression produced the corresponding effects: A20 overexpression reduced, while A20 knockdown increased the protein levels of IKK‐β, p‐IKK‐α/β, p‐IκBα, p‐NF‐κB‐p65, and BCL‐2, without altering IKK‐α levels (Figures 4F; S3B, Supporting Information). Moreover, overexpression of IKK‐β restored p‐NF‐κB‐p65 and BCL‐2 expression in A20‐overexpressed HCT116/L and THC8307/L cells, indicating that IKK‐β can rescue the A20‐mediated suppression of the p‐NF‐κB‐p65/BCL‐2 axis (Figure 4G). Together, these results demonstrate that IKK‐β is a critical upstream regulator through which A20 modulates NF‐kB‐p65 and BCL‐2 expression.

To investigate the impact of IKK‐β manipulation on oxaliplatin sensitivity, we generated IKK‐β overexpression and knockdown cell lines from both oxaliplatin‐sensitive and oxaliplatin‐resistant CRC cells (Figure S3C,D, Supporting Information). As expected, IKK‐β overexpression increased, whereas IKK‐β knockdown decreased the levels of p‐IKK‐α/β, p‐IκBα, p‐NF‐κB‐p65, and BCL‐2 (Figures 4H; S3E, Supporting Information). Functionally, IKK‐β knockdown sensitized cells to oxaliplatin, while IKK‐β overexpression promoted resistance, as demonstrated by cytotoxicity assays, colony formation assays, and Annexin V/PI staining (Figure S4, Supporting Information). Consistent with these findings, pharmacological inhibition using the IKK‐β inhibitor BMS‐345541 or the NF‐κB inhibitor Bay 11‐7082 significantly reversed oxaliplatin resistance induced by A20 deficiency (Figure S5, Supporting Information).

To evaluate the role of IKK‐β in vivo, BALB/c nude mice bearing subcutaneous CRC xenografts were assigned to four oxaliplatin‐treated groups: HCT116/THC8307‐OE‐VC, HCT116/THC8307‐OE‐IKK‐β, HCT116/L/THC8307/L‐shNC, and HCT116/LTHC8307/L‐shIKK‐β. The in vivo results mirrored those observed in vitro. IKK‐β knockdown enhanced tumor regression following oxaliplatin treatment, whereas IKK‐β overexpression diminished oxaliplatin sensitivity (Figures 4I–K; S6A–C, Supporting Information). Furthermore, Ki67 positivity and the levels of p‐IKK‐α/β, p‐NF‐κB‐p65, and BCL‐2, were reduced in xenografts with IKK‐β knockdown, while the opposite pattern was observed in the IKK‐β overexpression group (Figures 4L,M; S6D,E, Supporting Information). Together, these findings indicate that dysregulation of the A20/IKK‐β/NF‐κB/BCL‐2 pathway contributes to oxaliplatin resistance in CRC.

### 2.4 A20 Promotes the Ubiquitylation and Degradation of lKK‐β, Which is Essential for NF‐κB Activation

**Figure 5:**
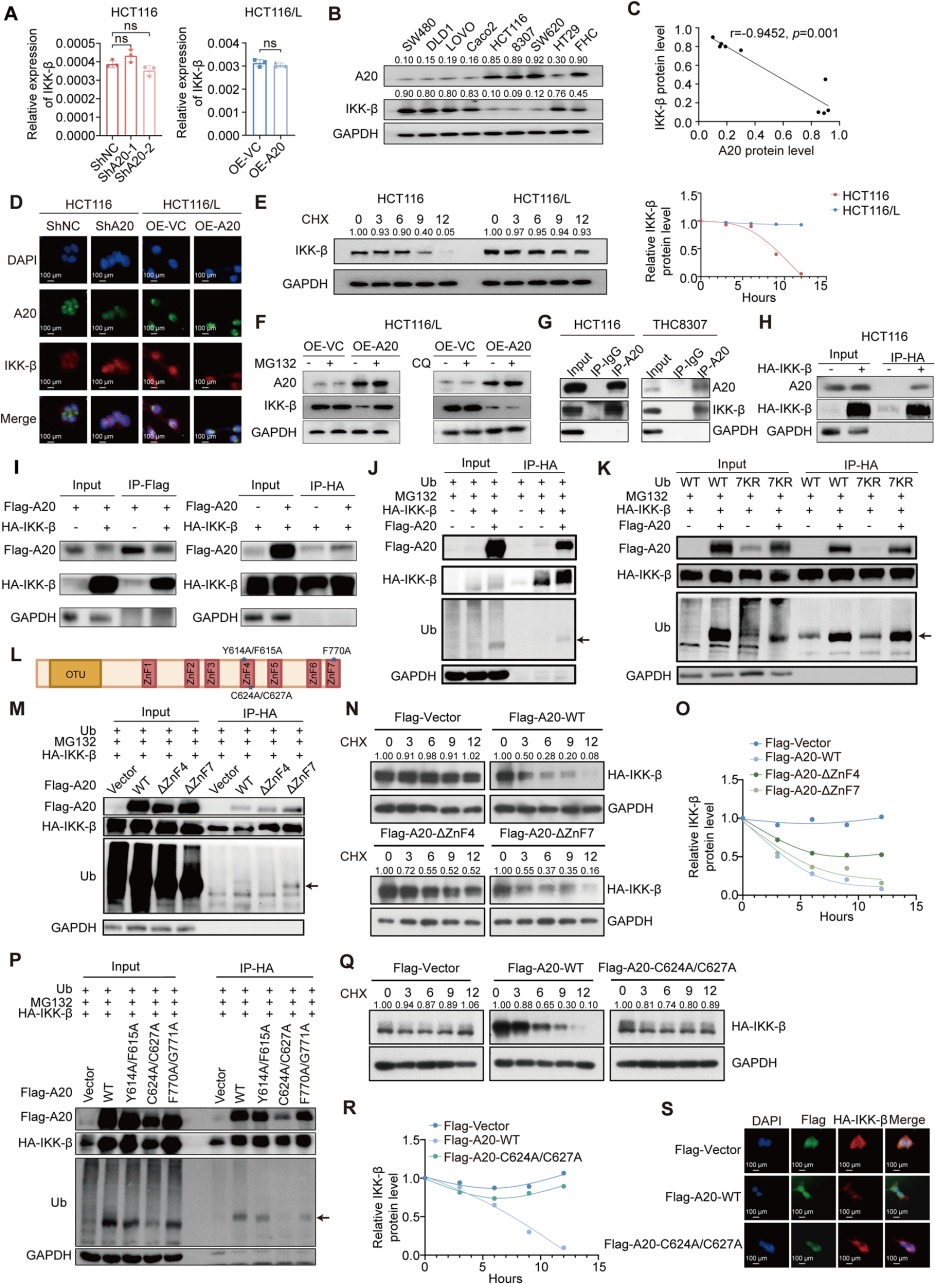
C624/C627 in the ZnF4 domain of A20 induces the monoubiquitylation of IKK‐β.A). mRNA expression levels of IKK‐β in A20 knockdown HCT116 cells and A20 overexpressing HCT116/L cells (n = 3).B). Western blot analysis of A20 and IKK‐β expression in representative CRC cell lines.C). Correlation between relative A20 and IKK‐β protein levels in representative CRC cell lines (n = 9).D). Representative immunofluorescence images showing DAPI, A20, and IKK‐β staining in HCT116‐shNC, HCT116‐shA20, HCT116/L‐OE‐VC, and HCT116/L‐OE‐A20 cells.E). Western blot analysis and relative protein levels of IKK‐β expression in HCT116 and HCT116/L cells treated with CHX (50 µM) for the indicated time points.F). Western blot analysis of A20 and IKK‐β expression in HCT116/L cells with or without A20 overexpression, treated with or without MG132 (20 µM) or chloroquine (CQ, 40 µM).G). Immunoprecipitation followed by western blot analysis of endogenous A20–IKK‐β interaction in HCT116 and THC8307 cells.H). Immunoprecipitation followed by western blot analysis of the interaction between HA‐IKK‐β and A20 in HCT116 cells.I). Immunoprecipitation followed by western blot analysis of HA‐IKK‐β and Flag‐A20 interaction in HEK293T cells.J). Immunoprecipitation followed by western blot analysis of HA‐IKK‐β interaction with Flag‐A20 or ubiquitin in HEK293T cells with or without A20 overexpression, treated with MG132 (20 µM).K). Immunoprecipitation followed by western blot analysis of HA‐IKK‐β ubiquitination (wild‐type or 7KR mutant ubiquitin) with or without A20 overexpression, treated with MG132 (20 µM).L). Diagram of A20 protein structure showing the OTU domain and seven ZnF domains. Asterisks (*) denote mutation sites.M). Immunoprecipitation followed by western blot analysis of HA‐IKK‐β interaction with Flag‐A20 (WT, ΔZnF4, ΔZnF7) or ubiquitin in HEK293T cells, treated with MG132 (20 µM).N). Western blot analysis of IKK‐β expression in HEK293T cells co‐transfected with HA‐IKK‐β and Flag‐Vector, Flag‐A20‐WT, Flag‐A20‐ΔZnF4, or Flag‐A20‐ΔZnF7, treated with CHX (50 µM) for the indicated time points.O). Relative IKK‐β protein levels corresponding to panel N.P). Immunoprecipitation followed by western blot analysis of HA‐IKK‐β interaction with Flag‐A20 (WT, Y614A/F615A, C624A/C627A, F770A/G771A) or ubiquitin in HEK293T cells, treated with MG132 (20 µM).Q). Western blot analysis of IKK‐β expression in HEK293T cells co‐transfected with HA‐IKK‐β and Flag‐Vector, Flag‐A20‐WT, or Flag‐A20‐C624A/C627A, treated with CHX (50 µM) for the indicated time points.R). Relative IKK‐β protein levels corresponding to panel Q.S). Representative immunofluorescence staining of DAPI, Flag, and HA‐IKK‐β in HEK293T cells co‐transfected with HA‐IKK‐β and Flag‐Vector, Flag‐A20‐WT, or Flag‐A20‐C624A/C627A.Data are presented as mean ± SD; ns, no significance, ^*^ *p *< 0.05, ^**^ *p* < 0.01, ^***^ *p* < 0.001.

To determine how A20 regulates lKK‐β, we first examined lKK‐β mRNA levels in parental and oxaliplatin‐resistant CRC cells. No significant differences in lKK‐β transcript levels were observed following A20 knockdown or overexpression (**Figures** **5**A; S7A, Supporting Information). In contrast, A20 protein levels were negatively correlated with lKK‐β protein abundance (Figure 5B,C). Immunofluorescence staining further showed that A20 and lKK‐β colocalize in the cytoplasm. Consistently, A20 knockdown in oxaliplatin‐sensitive CRC cells increased IKK‐β protein expression, whereas A20 overexpression in resistant cells reduced IKK‐β levels (Figures 5D; S7B, Supporting Information).

To evaluate whether A20 promotes lKK‐β degradation, we performed cycloheximide (CHX) chase assays. CHX treatment markedly reduced IKK‐β protein levels in oxaliplatin‐sensitive cells, whereas no significant degradation was observed in oxaliplatin‐resistant cells (Figures 5E; S7C,D, Supporting Information). Moreover, the proteasome inhibitor MG132 restored IKK‐β stability in A20‐overexpressing cells, while the lysosomal inhibitor chloroquine had no effect, indicating that A20 induces IKK‐β degradation through the UPS (Figures 5F; S7E, Supporting Information).

To further investigate how A20 dysfunction contributes to oxaliplatin resistance, we examined the interaction between A20 and IKK‐β. Immunoprecipitation of endogenous proteins, as well as co‐immunoprecipitation assays, demonstrated that A20 specifically binds to IKK‐β (Figure 5G–I). Given A20's established ubiquitin‐editing function, we next assessed the ubiquitylation status of IKK‐β. A20 overexpression in HEK293T cells significantly enhanced IKK‐β ubiquitination (Figure 5J). Pulldown assays using the His‐Ub7KR ubiquitin mutant–where all seven lysine residues were substituted with arginine–confirmed that IKK‐β undergoes monoubiquitylation (Figure 5K).

A20 contains an N‐terminal ovarian tumor domain with deubiquitinating activity and a C‐terminal ZnF domain with E3 ligase activity^[^ ^35^ ^]^ (Figure 5L). To identify which region mediates IKK‐β monoubiquitylation, we generated A20 mutants lacking specific ZnF motifs. Deletion of the ZnF4 domain substantially reduced IKK‐β monoubiquitylation, whereas ZnF7 deletion had no effect (Figure 5M). Consistently, CHX chase assays showed that disruption of ZnF4 impaired A20‐mediated IKK‐β degradation, underscoring the essential role of ZnF4 in this process (Figure 5N,O).

We further examined the physiological role of the ZnF4 motif by testing ZnF4‐specific mutations. The C624A/C627A mutation markedly reduced IKK‐β monoubiquitylation, whereas other mutations within the ZnF region (F770A/G771A and Y614A/F615A) had no significant effect (Figure 5P). Consistent with this, CHX chase assays showed increased IKK‐β stability in the presence of the C624A/C627A mutant (Figure 5Q,R). Immunofluorescence staining also demonstrated that A20 reduced IKK‐β expression, but this reduction was abolished by the C624A/C627A mutation (Figure 5S). Together, these findings confirm that the C624A/C627A mutation within ZnF4 disrupts A20‐mediated monoubiquitylation and degradation of IKK‐β, underscoring the essential function of the ZnF4 motif.

### 2.5 Monoubiquitylation of the lKK‐β Lysine Residue K163 is Critical for Oxaliplatin Resistance

**Figure 6:**
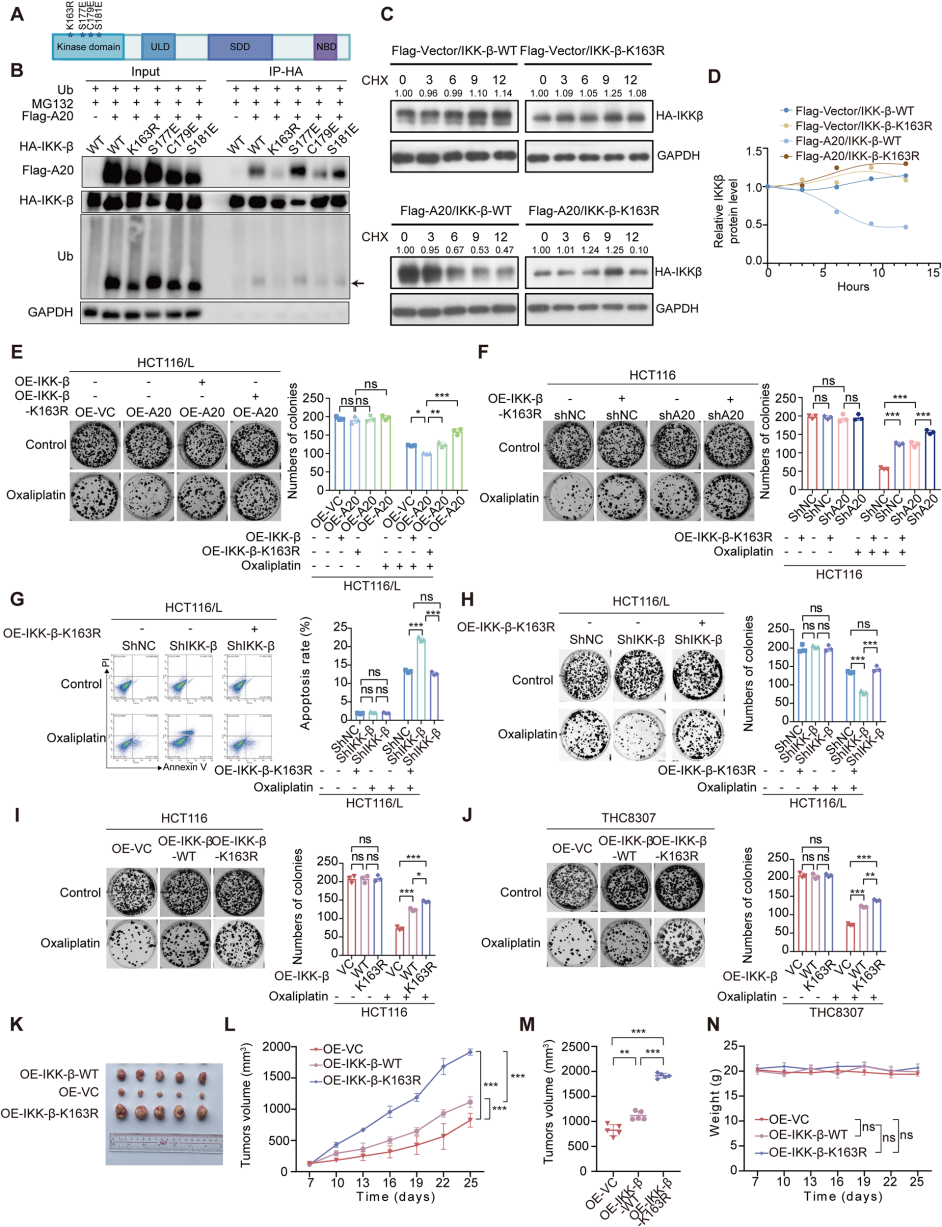
The lysine residue K163 of IKK‐β monoubiquitylated by A20 is critical for oxaliplatin resistance.A). Diagram of the IKK‐β protein structure, showing kinase domain, ubiquitin‐like domain (ULD), scaffold dimerization domain (SDD) and NEMO‐binding domain (NBD). Asterisks (*) indicate mutation sites.B). Immunoprecipitation followed by western blot analysis of the interaction between Flag‐tagged‐A20 (Flag‐A20) or ubiquitin with HA‐tagged‐IKK‐β (WT), HA‐tagged‐K163R mutant IKK‐β (K163R), HA‐tagged‐S177E mutant IKK‐β (S177E), HA‐tagged‐C179E mutant IKK‐β (C179E), or HA‐tagged‐S181E mutant IKK‐β (S181E) in HEK293T cells.C). Western blot analysis of IKK‐β protein stability in HEK293T cells co‐transfected with either HA‐IKK‐β WT or HA‐IKK‐β K163R and Flag‐Vector or Flag‐A20, treated with CHX (50 µM) for the indicated time points.D). Relative IKK‐β protein levels corresponding to panel C.E). Representative images and quantification of colony formation in HCT116/L OE‐VC and HCT116/L‐OE‐A20 cells with or without overexpression of WT or K163R IKK‐β, treated with control or oxaliplatin (n = 3).F). Representative images and quantification of colony formation in HCT116‐shNC and HCT116‐shA20 cells with or without K163R IKK‐β overexpression, treated with control or oxaliplatin (n = 3).G). Representative images and quantification of annexin V/propidium iodide staining in HCT116/L shNC and HCT116/L‐shIKK‐β cells with or without K163R mutant IKK‐β overexpression, treated with control or oxaliplatin (n = 3).H). Representative images and quantification of colony formation in HCT116/L‐shNC and HCT116/L‐shIKK‐β cells with or without K163R IKK‐β overexpression, treated with control or oxaliplatin (n = 3).I). Representative images and quantification of colony formation in HCT116‐OE‐VC, HCT116‐OE‐IKK‐β WT, and HCT116‐OE‐IKK‐β K163R cells treated with control or oxaliplatin (n = 3).J). Representative images and quantification of colony formation in THC8307‐OE‐VC, THC8307‐OE‐IKK‐β WT, and THC8307‐OE‐IKK‐β K163R cells treated with control or oxaliplatin (n = 3).K). Representative tumor images from BALB/c nude mice bearing THC8307 xenografts treated with oxaliplatin in the OE‐VC, OE‐IKK‐β WT, and OE‐IKK‐β K163R groups (n = 5).L‐N). Tumor outgrowth (L), tumor volume at the final time point (M), and body weight (N) of oxaliplatin‐treated mice in the OE‐VC, OE‐IKK‐β WT, and OE‐IKK‐β K163R groups (n = 5).Data are presented as mean ± SD; ns, no significance, ^*^ *p *< 0.05, ^**^ *p* < 0.01, ^***^ *p* < 0.001.

To identify the residues required for A20‐mediated monoubiquitylation of IKKβ, we generated four point mutations (K163R, S177E, C179E, and S181E) based on predictions from ubiquitination databases (UbiNet 2.0: https://awi.cuhk.edu.cn/~ubinet/search_v2.php?search_type=keyword; Uniprot: https://www.uniprot.org). Pulldown assays revealed that the IKK‐β K163R mutant could not undergo A20‐mediated monoubiquitylation (**Figure** **6**A,B). Consistently, CHX chase assays showed increased stability of the IKK‐β K163R mutant in the presence of A20 (Figure 6C,D). Functional assays further demonstrated that both IKK‐β overexpression and expression of the IKK‐β K163R mutant reversed the A20 overexpression‐induced increase in oxaliplatin sensitivity (Figures 6E; S8A, Supporting Information). Similarly, overexpression of the IKK‐β K163R mutant in wild‐type or A20‐knockdown HCT116/THC8307 cells further potentiated A20‐knockdown‐induced oxaliplatin resistance (Figures 6F; S8B, Supporting Information). Together, these findings indicate that A20 enhances oxaliplatin sensitivity in CRC cells through a mechanism dependent on IKK‐β monoubiquitylation at K163.

We next examined whether IKK‐β monoubiquitylation influences the cellular response to oxaliplatin. Re‐expression of the IKK‐β K163R mutant in resistant cells increased proliferation and reduced apoptosis (Figures 6G; S8C, Supporting Information). In colony formation assays, IKK‐β wild‐type (WT) significantly promoted CRC cell proliferation under oxaliplatin treatment, and this effect was further amplified by the IKK‐β K163R mutation (Figures 6H; S8D, Supporting Information).

To evaluate the impact of the IKK‐β K163R mutant on oxaliplatin resistance in vitro and in vivo, we first performed colony formation assays. IKK‐β WT significantly increased CRC cell proliferation under oxaliplatin treatment, and this effect was further enhanced by the lKK‐β K163R mutation (Figure 6I,J). We then established a subcutaneous xenograft model in BALB/c nude mice implanted with THC8307‐OE‐VC, THC8307‐OE‐IKK‐β WT, or THC8307‐lKK‐β K163R cells and treated the animals with oxaliplatin. The inhibitory effect of oxaliplatin on tumors derived from THC8307‐lKK‐β WT cells was markedly reduced compared with THC8307‐OE‐VC controls. Notably, oxaliplatin‐mediated tumor suppression was further diminished in the THC8307‐IKK‐β K163R group compared with the THC8307‐IKK‐β WT group (Figure 6K–N).

These findings underscore the essential role of the IKK‐β K163 residue in A20‐mediated IKK‐β degradation and the development of oxaliplatin resistance, suggesting that targeting the IKK‐β K163R mutation may represent a promising therapeutic strategy for overcoming oxaliplatin resistance in CRC.

### 2.6 Clinical Relevance of the A20/lKK‐β/p‐NF‐κB‐p65/BCL‐2 Axis in CRC Patients

**Figure 7:**
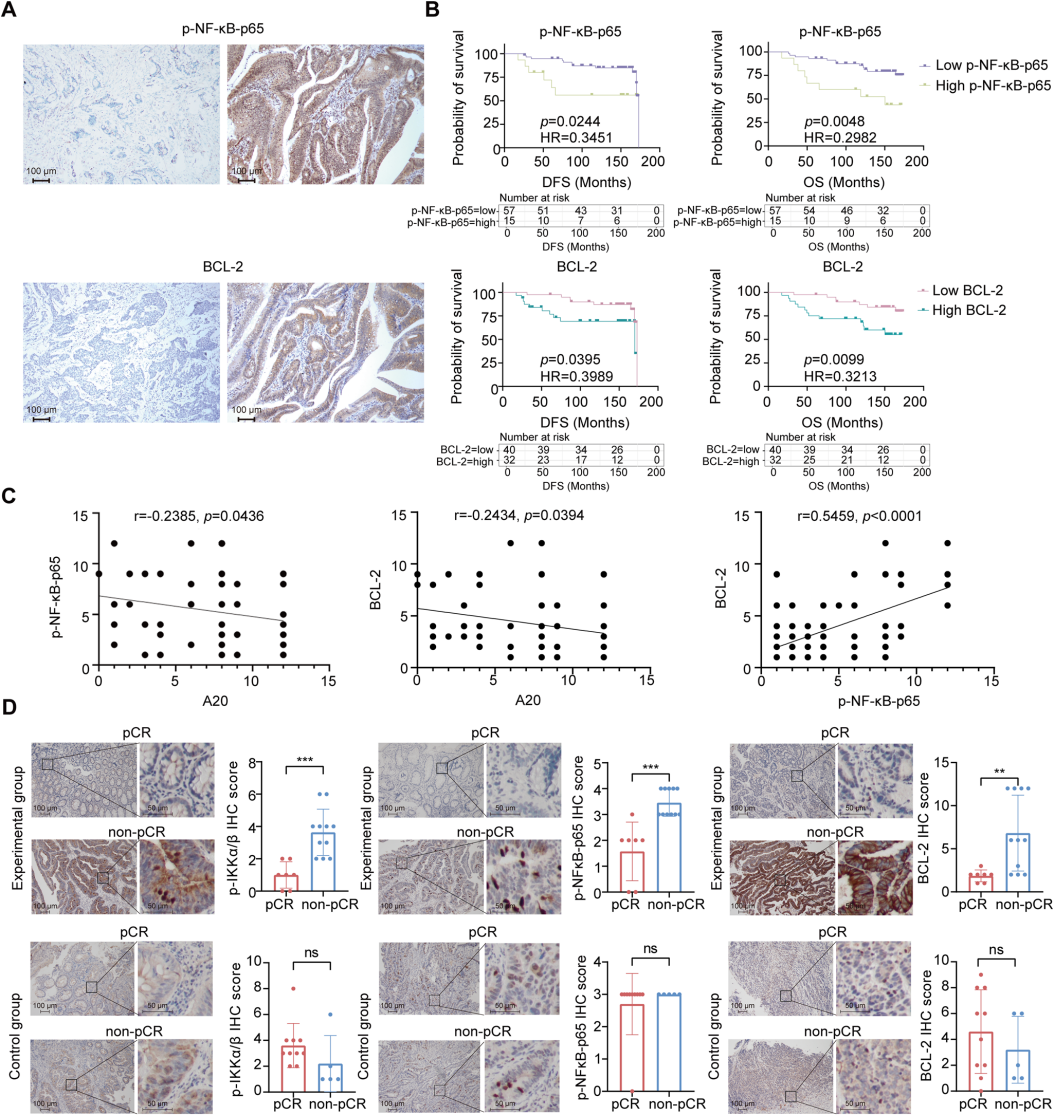
A20 restrains the lKK‐β/p‐NF‐κB‐p65/BCL‐2 axis in CRC patients.A). Representative immunohistochemical staining images of p‐NF‐κB‐p65 and BCL‐2 in CRC patients who received radical surgery followed by FOLFOX adjuvant chemotherapy in cohort II.B). Kaplan‒Meier analysis of DFS and OS in CRC patients who received radical surgery following FOLFOX adjuvant chemotherapy in cohort II, based on the protein levels of p‐NF‐κB‐p65 (Low p‐NF‐κB‐p65, n = 57; High p‐NF‐κB‐p65, n = 15) and BCL‐2 (Low BCL‐2, n = 40; High BCL‐2, n = 32).C). Spearman correlation analysis of A20 and p‐NF‐κB‐p65 expression, A20 and BCL‐2 expression, and BCL‐2 and p‐NF‐κB‐p65 expression in CRC patients who received radical surgery followed by FOLFOX adjuvant chemotherapy in cohort II (n = 72).D). Representative images and quantification of immunohistochemical staining for p‐IKKα/β, p‐NF‐κB‐p65, and BCL‐2 in pCR and non‐pCR groups of CRC patients who received oxaliplatin plus capecitabine neoadjuvant chemoradiotherapy (experimental group: pCR, n = 7; non‐pCR, n = 11) or capecitabine neoadjuvant chemoradiotherapy (control group: pCR, n = 10; non‐pCR, n = 5) in cohort I).Data are presented as mean ± SD; ns, no significance, ^*^ *p *< 0.05, ^**^ *p* < 0.01, ^***^ *p* < 0.001.

Given the above results, we next evaluated the expression of A20, p‐NF‐κB‐p65, and BCL‐2 in tumor tissues from CRC patients who underwent radical surgery following FOLFOX adjuvant chemotherapy. Histological and immunohistochemical analyses showed that patients with higher levels of p‐NF‐κB‐p65 (*p* = 0.0244, HR = 0.3451 (95% CI, 0.0937‐1.272); *p* = 0.0048, HR = 0.2922 (95% CI, 0.0919‐0.9689)) and BCL‐2 (*p* = 0.0395, HR = 0.3989 (95% CI, 0.1496‐1.063); *p* = 0.0099, HR = 0.3213 (95% CI, 0.1299‐0.7950)) exhibited significantly shorter DFS and OS compared with patients with low expression of these proteins (**Figure** **7**A,B). As expected, p‐NF‐κB‐p65 and BCL‐2 expression negatively correlated with A20 expression, while BCL‐2 expression positively correlated with p‐NF‐κB‐p65 levels (Figure 7C). These data suggest that p‐NF‐κB‐p65 and BCL‐2 dysregulation in CRC is driven, at least in part, by the loss of A20.

We further examined whether p‐IKK‐α/β, p‐NF‐κB‐p65, and BCL‐2 could serve as biomarkers of oxaliplatin response. IHC staining of paired preoperative biopsy and surgical specimens from CRC patients revealed that non‐pCR patients displayed higher levels of these markers than pCR patients in the experimental (oxaliplatin‐treated) group (Figure 7D). In contrast, no significant differences were observed between pCR and non‐pCR patients in the control group (Figure 7D). These findings indicate that the A20/IKK‐β/p‐NF‐κB‐p65/BCL‐2 axis plays a critical role in determining oxaliplatin response and may serve as a biomarker panel for predicting oxaliplatin efficacy.

Collectively, through analyses of a Phase III cohort of LARC patients treated with oxaliplatin‐based or non‐oxaliplatin neoadjuvant chemoradiotherapy, and an independent cohort of 100 CRC patients receiving FOLFOX or FOLFIRI adjuvant chemotherapy, we identified loss of A20 as a key driver of oxaliplatin resistance. Mechanistically, we demonstrate that A20 binds to IKK‐β via its ZnF4 domain, promoting monoubiquitylation of IKK‐β K163 and suppressing activation of the IKK‐β/NF‐κB/BCL‐2 axis. This work identifies A20 as a previously unrecognized mediator of oxaliplatin resistance and provides a potential therapeutic strategy for oxaliplatin‐resistant CRC.

## 3 Discussion

Oxaliplatin resistance remains a major clinical obstacle in the management of CRC. Previous studies have highlighted the involvement of the UPS in tumor progression and chemoresistance.^[^ ^36^ ^]^ In the present study, using a Phase III clinical cohort of locally advanced CRC patients receiving oxaliplatin‐based or non‐oxaliplatin neoadjuvant chemoradiotherapy, together with additional independent patient cohorts, we identified A20 inactivation as a key determinant of reduced oxaliplatin response and poor prognosis. Mechanistically, A20 loss promotes oxaliplatin resistance through activation of the IKK‐β‐mediated NF‐κB/BCL‐2 pathway. We demonstrate that A20 directly interacts with IKK‐β and induces its degradation through monoubiquitylation. This regulatory effect is abolished by deletion of the A20 ZnF4 domain or by substitution of IKK‐β lysine 163 (K163R). Both in vitro and in vivo models confirm that inhibition of IKK‐β restores sensitivity to oxaliplatin. Collectively, these findings establish a functional link between A20 deficiency and oxaliplatin resistance and provide a strong preclinical rationale for targeting IKK‐β as a therapeutic strategy for patients with oxaliplatin‐resistant CRC.

Our findings identify a tumor‐suppressive role for A20 in CRC. A20 was originally described as a tumor suppressor in B cell lymphomas,^[^ ^37^ ^]^ however, accumulating evidence shows that A20 exerts context‐dependent and often opposing functions in solid tumors. In CRC and hepatocellular carcinoma, A20 suppresses tumor progression,^[^ ^38^, ^39^ ^]^ whereas in breast cancer, gastric cancer, and melanoma, it functions as an oncogene.^[^ ^40^, ^41^, ^42^ ^]^ Despite its recognized involvement in anti‐cancer drug resistance,^[^ ^43^ ^]^ the role of A20 varies by cancer type and therapeutic agent.^[^ ^18^, ^20^, ^44^ ^]^ Several studies report that A20 overexpression contributes to resistance to endocrine therapy, chemotherapy, and radiotherapy; for example, tamoxifen‐resistant breast cancer cells exhibit elevated A20 levels.^[^ ^21^ ^]^ However, the relationship between A20 expression and oxaliplatin resistance has remained unclear. In our study, we observed that A20 levels were significantly reduced in oxaliplatin‐resistant CRC cells compared with oxaliplatin‐sensitive cells. Clinically, CRC patients with higher A20 expression showed better responses to oxaliplatin‐based chemotherapy. These findings expand our current understanding by demonstrating that A20 dysfunction contributes directly to oxaliplatin resistance in CRC.

Furthermore, our study provides novel mechanistic insights into how A20 downregulation drives oxaliplatin resistance. We show that low A20 expression promotes resistance through activation of anti‐apoptotic signaling, particularly via upregulation of BCL‐2. Manipulating BCL‐2 levels effectively altered oxaliplatin‐induced apoptosis in CRC cell lines, and in vivo models confirmed that A20 suppresses BCL‐2 expression. BCL‐2 is widely recognized for its role in mediating resistance to cytotoxic therapies, and recent work has suggested that BCL‐2 upregulation in CRC may predict poor outcomes in patients receiving oxaliplatin‐based chemotherapy^[^ ^45^ ^]^ – which is consistent with our findings. In contrast, Chen et al. reported that A20 silencing induced apoptosis by downregulating BCL‐2 expression in Jurkat and Reh cells.^[^ ^22^ ^]^ These discordant results highlight the context‐dependent nature of A20, which can exhibit pro‐ or anti‐apoptotic functions depending on the cellular environment and therapeutic context. Our data indicate that, in CRC under oxaliplatin treatment, A20 promotes apoptosis by suppressing BCL‐2 expression. Future studies should investigate whether pharmacological inhibition of BCL‐2 could help overcome oxaliplatin resistance in patients with low A20 expression.

Given that BCL‐2 is a critical downstream effector of the NF‐κB pathway,^[^ ^46^ ^]^ we next examined whether A20 dysfunction upregulates BCL‐2 by activating NF‐κB signaling. Our results demonstrate that A20 deficiency enhances NF‐κB activity and increases BCL‐2 expression, consistent with previous studies showing that A20 is a potent suppressor of NF‐κB signaling. In mouse models, A20 loss leads to excessive NF‐κB activation and multiorgan inflammation.^[^ ^13^, ^23^ ^]^ Similarly, in ulcerative colitis, A20 deficiency results in marked upregulation of NF‐κB‐p65 and its downstream targets – including inflammatory mediators and anti‐apoptotic factors – which together may contribute to a tumor‐promoting microenvironment.^[^ ^47^, ^48^ ^]^

A20 contains an N‐terminal deubiquitinating OTU domain and multiple C‐terminal zinc‐finger motifs, which together regulate ubiquitination at the level of NEMO, a core component of the IκB kinase (IKK) complex. However, direct evidence of A20's inhibitory effects on IKK activation has remained limited. One study reported that A20 can directly suppress IKK activation independently of its deubiquitinase or E3 ligase activities; specifically, polyubiquitin binding by the A20 ZnF7 motif facilitates selective interaction with NEMO, thereby impairing IKK phosphorylation.^[^ ^17^ ^]^

A20 regulates diverse cellular processes through both polyubiquitination and monoubiquitylation. Beyond its established roles in mediating the polyubiquitination and degradation of proteins such as NEK7, C/EBPβ, and KEAP1,^[^ ^49^, ^50^, ^51^ ^]^ our study demonstrates that A20 promotes oxaliplatin sensitivity in CRC by inducing monoubiquitylation of IKK‐β. This finding significantly extends the known scope of A20‐mediated monoubiquitination, which was previously implicated in promoting metastasis in basal‐like breast cancer via Snail modification.^[^ ^40^ ^]^

Other studies have highlighted the importance of IKK‐β ubiquitylation– mediated by distinct E3 ligases–in regulating NF‐κB signaling. For instance, USP5 and pVHL promote IKK‐β polyubiquitylation, thereby modulating IKK/NF‐κB pathway activation,^[^ ^52^, ^53^ ^]^ whereas the RING‐finger protein Ro52 downregulates Tax‐induced NF‐κB signaling by monoubiquitylating IKK‐β.^[^ ^54^ ^]^ Together with these findings, our data support the view that IKK‐β ubiquitination is essential for proper NF‐κB pathway regulation. Importantly, we provide novel evidence that A20 interacts with the K163 lysine residue of IKK‐β, a component of the IKK complex, to restrain NF‐κB signaling by promoting monoubiquitylation‐dependent degradation of IKK‐β. These observations align with recent work demonstrating that A20‐ZnF4‐mediated degradation of Ubc13 and UbcH5C suppresses NF‐κB signaling,^[^ ^55^ ^]^ further underscoring the essential function of A20 ZnF4 in NF‐kB pathway regulation.

Further studies have reported that gefitinib‐resistant head and neck squamous cell carcinoma (HNSCC) cells exhibit upregulation of the IKK‐β/NF‐κB signaling pathway, and that the combined targeting of EGFR and IKK‐β may provide a promising therapeutic strategy for HNSCC.^[^ ^56^ ^]^ A similar association between gefitinib resistance and dysregulated IKK‐β/NF‐κB signaling has been observed in non‐small cell lung cancer.^[^ ^57^ ^]^ These findings suggest that inhibition of the IKK‐β/NF‐κB pathway could be an effective approach to reverse drug resistance, potentially explaining the enhanced oxaliplatin sensitivity we observed following genetic suppression of IKK‐β.

Numerous IKK‐β/NF‐κB inhibitors have entered preclinical development, demonstrating robust anti‐tumor activity and the ability to overcome drug resistance in several solid tumor models. For example, BMS‐345541, a highly selective IKK‐β inhibitor, effectively induces apoptosis in melanoma cells and markedly enhances their sensitivity to doxorubicin.^[^ ^58^ ^]^ Likewise, JSH‐23, an inhibitor of NF‐κB transcriptional activity, suppresses the survival and metastatic capacity of lung adenocarcinoma cells and resensitizes resistant esophageal squamous cell carcinoma cells to linsitinib.^[^ ^59^, ^60^ ^]^ Beyond these preclinical agents, several clinically used drugs with IKK‐β/NF‐κB inhibitory activity have been widely studied across various cancer types.^[^ ^61^ ^]^ Anti‐inflammatory agents such as sodium salicylate and aspirin demonstrate anti‐tumor effects in hematologic malignancies and solid tumors by blocking IκBα degradation and directly targeting IKK‐β to prevent NF‐κB activation.^[^ ^62^, ^63^ ^]^ Additionally, the proteasome inhibitor bortezomib–FDA‐approved for multiple myeloma–induces apoptosis in breast, prostate, ovarian, and pancreatic cancers by inhibiting NF‐κB signaling^[^ ^64^, ^65^, ^66^ ^]^ and can resensitize breast cancer cells to chemotherapy through modulation of multidrug resistance protein 1 (MDR1).^[^ ^67^ ^]^ Together, our findings support the therapeutic potential of IKK‐β/NF‐κB inhibitors as a safe and effective strategy for CRC patients with oxaliplatin resistance.

In conclusion, A20 represents a previously unrecognized vulnerability in oxaliplatin‐resistant CRC. Both in vitro and in vivo findings demonstrate that A20 downregulation promotes tumor proliferation and enhances resistance to oxaliplatin, whereas A20 overexpression suppresses tumor growth and increases apoptosis. Mechanistically, A20 exerts its function in CRC by monoubiquitylating IKK‐β at K163, leading to IKK‐β degradation and subsequent suppression inhibition of the NF‐κB/BCL‐2 signaling axis. Clinically, CRC patients with reduced A20 expression–or with elevated levels of p‐IKK‐β, p‐NF‐κB‐p65, and BCL‐2–exhibit poorer responses to oxaliplatin‐based therapy. Collectively, these results highlight the central role of the A20/IKK‐β/NF‐κB/BCL‐2 axis in mediating oxaliplatin resistance and support its potential as a promising therapeutic target for overcoming chemoresistance in CRC.

## 4 Experimental Section

### Cell Lines and Reagents

HCT116 (RRID: CVCL_0291) and HEK293T (RRID: CVCL_0063) cells were obtained from the American Type Culture Collection (ATCC, USA). The highly differentiated human colon adenocarcinoma cell line THC8307 (Cat# CL0854) was purchased from Fenghui Biotechnology (China). All cell lines were authenticated by short tandem repeat (STR) profiling and confirmed to be mycoplasma‐free.

Oxaliplatin‐resistant CRC cell lines (THC8307/L and HCT116/L) were generated by gradually increasing oxaliplatin concentrations over a 6‐month period using a stepwise escalation protocol:^[^ ^68^ ^]^ Cells were maintained in RPMI‐1640 medium (CRC cell lines) or DMEM (HEK293T) supplemented with 10% fetal bovine serum at 37 °C in a humidified atmosphere containing 5% CO_2_.

Oxaliplatin (Selleck Chemicals, USA) was prepared as a 10 mmol L^−1^ stock solution in double‐distilled water and stored at ‐20 °C. Z‐VAD‐FMK (Selleck Chemicals, USA), necrostatin‐1 (Selleck Chemicals, USA), ferrostatin‐1 (Selleck Chemicals, USA), disulfiram (Selleck Chemicals, USA), APG‐2575 (Selleck Chemicals, USA), BMS‐345541 (Selleck Chemicals, USA), Bay 11‐7082 (Selleck Chemicals, USA) was prepared as a 10 mmol L^−1^ stock solution in dimethyl sulfoxide and stored at ‐20 °C. For in vivo experiments, oxaliplatin was freshly diluted in 5% glucose prior to administration.

### Patient Information and Tissue Specimens

Paraffin embedded and frozen CRC tissue samples were collected from patients diagnosed by pathological and clinical evaluation at Sun Yat‐Sen University Cancer Center. Tumor tissues were used for IHC analyses.

Paired preoperative biopsy and surgical specimens from a Phase III randomized study of locally advanced rectal cancer (cohort I) were used to evaluate oxaliplatin response based on pathological tumor regression grade, and classified into four tiers according to Ryan's score.^[^ ^69^ ^]^ A score of 0 was defined as pathological complete regression (pCR), whereas scores 1‐3 were categorized as non‐pCR.^[^ ^70^, ^71^ ^]^

Cohort II consisted of 100 CRC patients who underwent radical surgery followed by FOLFOX or FOLFIRI adjuvant chemotherapy between 2008 and 2011 and were included for Kaplan‐Meier survival analysis.

Additionally, frozen tissues from 12 paired samples of CRC tumors (T) and adjacent normal tissues (N) were stored at −80 °C for Western blot analysis (cohort III).

### Immunohistochemistry Staining

Formalin‐fixed, paraffin‐embedded tissues from CRC patients and xenografted tumors from BALB/c nude mice were sectioned at 3 µm thickness. Antigen retrieval was performed by microwaving sections at 100 °C for 10 min in 0.1 M citric acid buffer (pH = 6.0), followed by overnight incubation with primary antibodies at 4 °C.

Primary antibodies included those targeting human A20 (1:100), human BCL‐2 (1:200), human phosphorylated (p)‐NF‐κB‐p65 (1:200), human p‐IKKα/β (1:100), murine Ki‐67 (1:500), murine A20 (1:100), murine BCL‐2 (1:500), murine p‐NF‐κB‐p65 (1:500), and murine p‐IKKα/β (1:100). Full antibody information is provided in Table S5 (Supporting Information).

After incubation with species‐appropriate secondary antibodies at room temperature for 1 h, staining was developed using 0.05% diaminobenzidine containing 0.01% hydrogen peroxide. For negative controls, normal goat serum was substituted for primary antibodies and slices were incubated overnight at 4 °C before proceeding with the staining protocol. Positive signals were visualized using a fluorescence microscope.

Immunostaining was quantified using an IHC score calculated as previously described.^[^ ^72^ ^]^ Two parameters were assessed: 1) the proportion of positively stained cells, scored as 0 (<5%), 1 (5–25%), 2 (26–50%), 3 (51–75%), or 4 (>75%), and 2) staining intensity, scored as 0 (no staining), 1 (faintly positive, +), 2 (moderately positive, ++), or 3 (strongly positive, +++). The composite IHC score (range 0–12) was obtained by multiplying these two values.

### Western Blot Analysis

Whole‐cell lysates were prepared using RIPA buffer (Santa Cruz Biotechnology, Germany), and protein concentrations were determined with the BCA Protein Assay Kit (Pierce Biotechnology, USA). Equal amounts of protein were separated on 10% SDS‐PAGE gels and transferred onto polyvinylidene difluoride membranes (Millipore, USA).

After blocking, membranes were incubated with primary antibodies against BCL‐2, XIAP, BAK, BCL‐XL, BAD, p‐NF‐κB‐p65, NF‐κB‐p65, p‐IKKα/β, IKK‐α, IKK‐β, IKK‐γ, IKK‐ε, p‐IKBα, IKBα, ubiquitin, β‐actin, histone, GAPDH, HA, Flag, and A20 (details in Table S5, Supporting Information). Following washes, membranes were incubated with horseradish peroxidase‐conjugated secondary antibodies (1:2500). Protein bands were visualized using enhanced chemiluminescence (Pierce ECL kit, Thermo Fisher Scientific, USA).

### Cell Transfection

To generate A20 stable overexpression or knockdown cell lines, the full‐length A20 coding sequence was PCR‐amplified and cloned into the pReceiver‐Lv181‐3XFlag vector. Short hairpin RNA targeting A20, along with a scrambled shRNA control (shNC), were cloned into the pSIH‐H1‐puro vector.

Lentiviral packaging was performed in HEK293T cells seeded at 80% confluency in 10‐cm dishes and transfected using Lipofectamine 3000 reagent (Life Technologies, USA), according to the manufacturer's instructions. Viral supernatants were collected and used to infect CRC cell lines, which were incubated overnight at 37 °C. After 48 h, puromycin (1 µg mL^−1^) was added for selection to establish stable cell lines.

For lentiviral infections, CRC cells were transduced with viral particles encoding scrambled control, BCL‐2, IKK‐β‐wild type, IKK‐β‐K163R, shNC, shBCL‐2, shIKK‐β#1, shIKK‐β#2, or shIKK‐β#3, followed by selection with puromycin (1 µg/mL) for 72 h. Stable BCL‐2 and IKK‐β (wild type/or K163R) overexpression and knockdown cell lines were verified by Western blot.

Transient overexpression was performed using polyethyleneimine (PEI) transfection. Plasmids used for both transient and stable transfections are listed in Table S5 (Supporting Information), and primer/oligonucleotide sequences are provided in Table S6 (Supporting Information).

### Cell Cytotoxic Assay

Cell cytotoxicity was assessed using the Cell Counting Kit‐8 (CCK‐8; Dojindo, CK04) to evaluate oxaliplatin sensitivity, following previously described protocols.^[^ ^73^ ^]^ Each experiment was performed in triplicate. The oxaliplatin concentration required to inhibit 50% of cell proliferation was calculated from dose‐response survival curves using the Bliss method and applied in subsequent experiments.

### Real‐Time Cell Analysis

Real‐time cell proliferation was monitored using the RTCA S16 system (ACEA Biosciences, San Diego, CA, USA). After obtaining the impedance background, CRC cells were seeded into E‐plates and treated with the indicated drugs. Plates were equilibrated for 30 min at room temperature and then placed in the RTCA station, where impedance was recorded every 15 min for over 100 h. Cell proliferation was quantified based on cell index values calculated by the RTCA software.

### Colony Formation Assay

CRC cell lines were treated with the BCL‐2 inhibitor APG‐2575, IKK‐β inhibitor BMS‐345541, NF‐κB inhibitor Bay 11‐7082 or oxaliplatin at the indicated concentrations for 2 days, and then trypsinized. A total of 1000 cells were seeded into six‐well plates (Corning‐Costar, 3516) and cultured for 2‐3 weeks, until visible colonies formed. Colonies were fixed and stained with 0.1% crystal violet, and colony numbers were subsequently counted. Each experiment was performed in triplicate.

### Flow Cytometry Analysis

For cell cycle analysis, cells were fixed in 70% ethanol for at least 2 h, treated with RNase A (100 µg/mL) for 10 min to remove RNA, and subsequently stained with propidium iodide. Apoptosis was assessed using Annexin V/PI double staining. Both cell cycle distribution and apoptosis were analyzed using a flow cytometer (BD, New Jersey, USA).

### Dual‐Luciferase Reporter Assay

Luciferase activity was measured using the Dual‐Luciferase Reporter Assay System (Promega, #E1910). Cells were seeded at 30% confluence in 24‐well plates and co‐transfected with a Renilla luciferase vector and either a BCL‐2 promoter‐luciferase reporter construct alone or the BCL‐2 promoter‐luciferase reporter together with pCMV‐RELA, as indicated. Transfections were performed using PEI. Firefly luciferase activity was normalized to Renilla luciferase activity to control for transfection efficiency.

### qRT‐PCR

Total RNA was extracted using TRIzol reagent (Invitrogen, 10 296 010), and cDNA was synthesized using a reverse transcription supermix kit (Bio‐Rad). Quantitative real‐time PCR was performed with SYBR Green Master Mix, and mRNA expression levels were normalized to the endogenous control ACTB (β‐Actin). Primer sequences were as follows:

ACTB‐F: CATGTACGTTGCTATCCAGGC

ACTB‐R: CTCCTTAATGTCACGCACGAT

IKK‐β‐F: CTGGCCTTTGAGTGCATCAC

IKK‐β‐R: CGCTAACAACAATGTCCACCT

### Immunofluorescence Analysis

Cells were fixed with 4% paraformaldehyde for 15 min, permeabilized with 0.2% Triton X‐100 for 30 min at room temperature, and washed three times with PBS. After blocking with 1% BSA for 30 min, cells were incubated with primary antibodies overnight at 4 °C. Secondary antibodies (Invitrogen, USA) were applied in the dark at 37 °C for 1 h, followed by staining with 4′,6‐diamidino‐2‐phenylindole (DAPI; 2 201 593, Invitrogen, USA) for 5 min. Images were captured using a fluorescence microscope (Olympus BX51).

### Ubiquitination Assay

Cells were treated with 50 µM CHX for the indicated time points (0, 3, 6, 9, and 12 h) or with 20 µM MG132 for 12 h. Cell lysates were collected, and A20/IKK‐β ubiquitination was analyzed by Western blot.

### Co‐Immunoprecipitation (Co‐IP) Assay

Protein lysates were prepared using IP lysis buffer (0.025 M Tris, pH 7.4; 0.15 M NaCl; 0.001 M EDTA; 1% NP40; 5% glycerol) and incubated overnight at 4 °C with anti‐Flag, anti‐HA, anti‐A20, or anti‐IgG antibodies. Immune complexes were captured using protein A/G magnetic beads for 10 min on ice, washed, and eluted with a low‐pH buffer. Eluted proteins were analyzed by Western blot using the Pierce Classic Magnetic IP/Co‐IP Kit (USA). Antibodies used are listed in Table S5 (Supporting Information).

### RNA‐seq and Gene Enrichment Analysis

Total RNA was extracted from oxaliplatin‐resistant and ‐sensitive CRC cells using TRIzol reagent. High‐quality RNA was used for sequencing library preparation, and RNA sequencing was performed on the Illumina NovaSeq platform. Gene expression levels were quantified as fragments per kilobase of exon per million mapped reads (FPKM). Differential gene expression analysis was conducted using EdgeR (http://www. bioconductor.org/packages/2.12/bioc/html/edgeR.html). Gene Ontology enrichment analysis was performed to identify significantly enriched biological pathways.

### Animal Experiments

Xenograft models were established using CRC cell lines. A total of 5 × 10^6^ HCT116 or THC8307 cells suspended in 0.1 mL PBS were subcutaneously injected into 6‐week‐old female BALB/c nude mice (five mice per group). When tumors reached ≈100 mm^3^, mice received intraperitoneal injections of oxaliplatin (10 mg kg^−1^) once weekly. Tumor growth was monitored every three days by measuring length and width with calipers, and tumor volume was calculated. After three weeks of treatment, mice were euthanized, and tumors were harvested, weighed, and processed for IHC analysis. All mice were obtained from Vital River Laboratory Animal Technology Co., Ltd. (Beijing, China) and housed in a controlled, pathogen‐free facility.

### Statistical Analysis

Statistical analyses were performed using IBM SPSS Statistics 19 (IBM Corp., Armonk, NY, USA) and GraphPad Prism v8.0 (GraphPad Software, USA). Survival curves were compared using the log‐rank test. Two‐tailed Student's t‐tests were used for comparisons between two groups, and one‐way ANOVA was used for comparisons involving three or more groups. Optimal cutoff values for A20 and IKK‐β expression were determined using the X‐Tile program following Camp et al.^[^ ^74^ ^]^ Data are presented as mean ± standard deviation from at least three independent experiments. A *p*‐value <0.05 was considered statistically significant.

### Ethics Approval

Written informed consent was obtained from all human participants. The study was approved by the Human Research Ethics Committee of Sun Yat‐sen University Cancer Center (SYSUCC; approval number B2022‐335‐01). All animal experiments were performed in accordance with the guidelines of the Institutional Animal Care and Use Committee of SYSUCC (approval number L102012023120K).

## Conflict of Interest

The authors declare no conflicts of interest.

## Author Contributions

F.L., T.Y., J.C., and Q.C. contributed equally to this work. F.L., W.J.M., M.L., and R.X.Z. contributed to the conceptualization and methodology of the study and provided supervision. Data curation was performed by F.L., T.Y., J.X.C., Q.C., Z.H.L., F.T.L., C.Z.L., Z.F.X., Y.Z.Y., and P.L. Formal analysis and the original draft were prepared by F.L., T.Y., J.X.C., and Q.C. The manuscript was reviewed and edited by F.L., W.J.M., M.L., and R.X.Z.

## Supporting information



Supporting Information

## Data Availability

The code and bulk RNA‐seq matrix for the study have been deposited in the Github (https://github.com/Zenphy/A20‐Facilitates‐Oxaliplatin‐Sensitivity‐in‐Colorectal‐Cancer‐through‐Monoubiquitylation‐of‐IKK‐git). The raw data of all the functional experiments of the main figures in this work has been deposited onto the Research Data Deposit public platform (www.researchdata.org.cn) with the approval number RDDB2025561993.
