# Supplementary material for: A20 Facilitates Oxaliplatin Sensitivity in Colorectal Cancer Through Monoubiquitylation of IKK‐β
Source: Adv Sci (Weinh). 2025 Dec 17;13(12):e14486. doi: 10.1002/advs.202514486 (PMC12948199; doi:10.1002/advs.202514486)
Supplement: Supplementary file 1 — Supporting Information [file ADVS-13-e14486-s001.pdf]

Supplementary Materials

Figure S1. Loss of A20 promotes oxaliplatin resistance *in vitro*

Figure S1

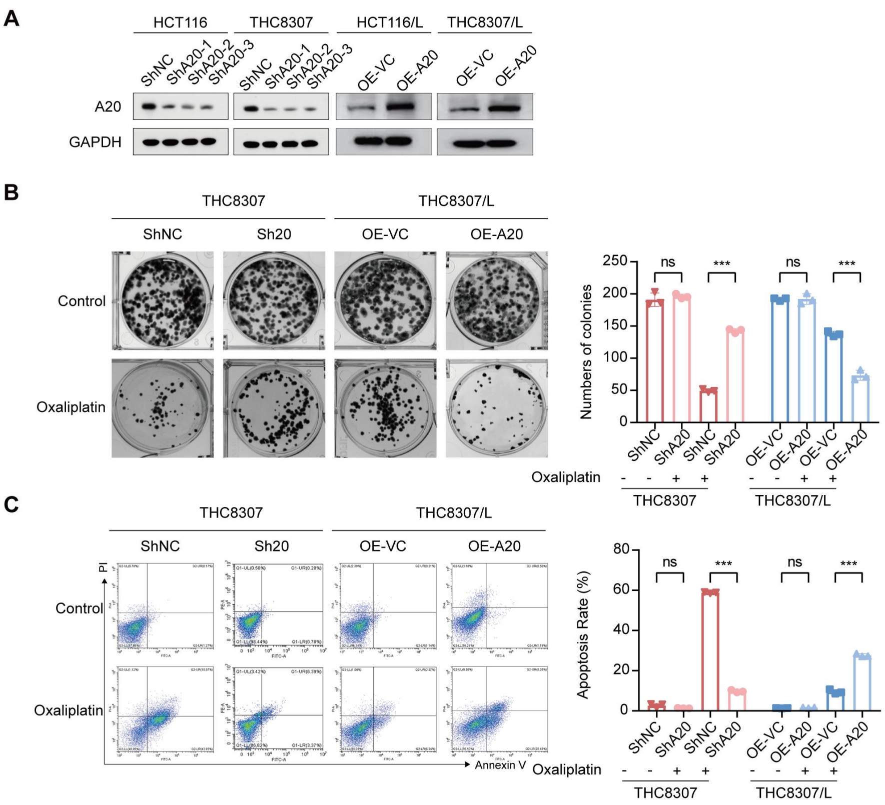

A. Western blot analysis showing the efficiency of A20 knockdown (shA20) and overexpression (OE-A20) in HCT116, THC8307, HCT116/L, and THC8307/L cells.

B. Representative images and quantification of colony formation in THC8307-shNC, THC8307-shA20, THC8307/L-OE-VC, and THC8307/L-OE-A20 cells treated with control or oxaliplatin (n = 3).

C. Representative images and quantification of annexin V/propidium iodide staining in THC8307-shNC, THC8307-shA20, THC8307/L-OE-VC, and THC8307/L-OE-A20 cells treated with control or oxaliplatin (n = 3).

Figure S2. BCL-2 activation leads to oxaliplatin resistance *in vitro*

Figure S2

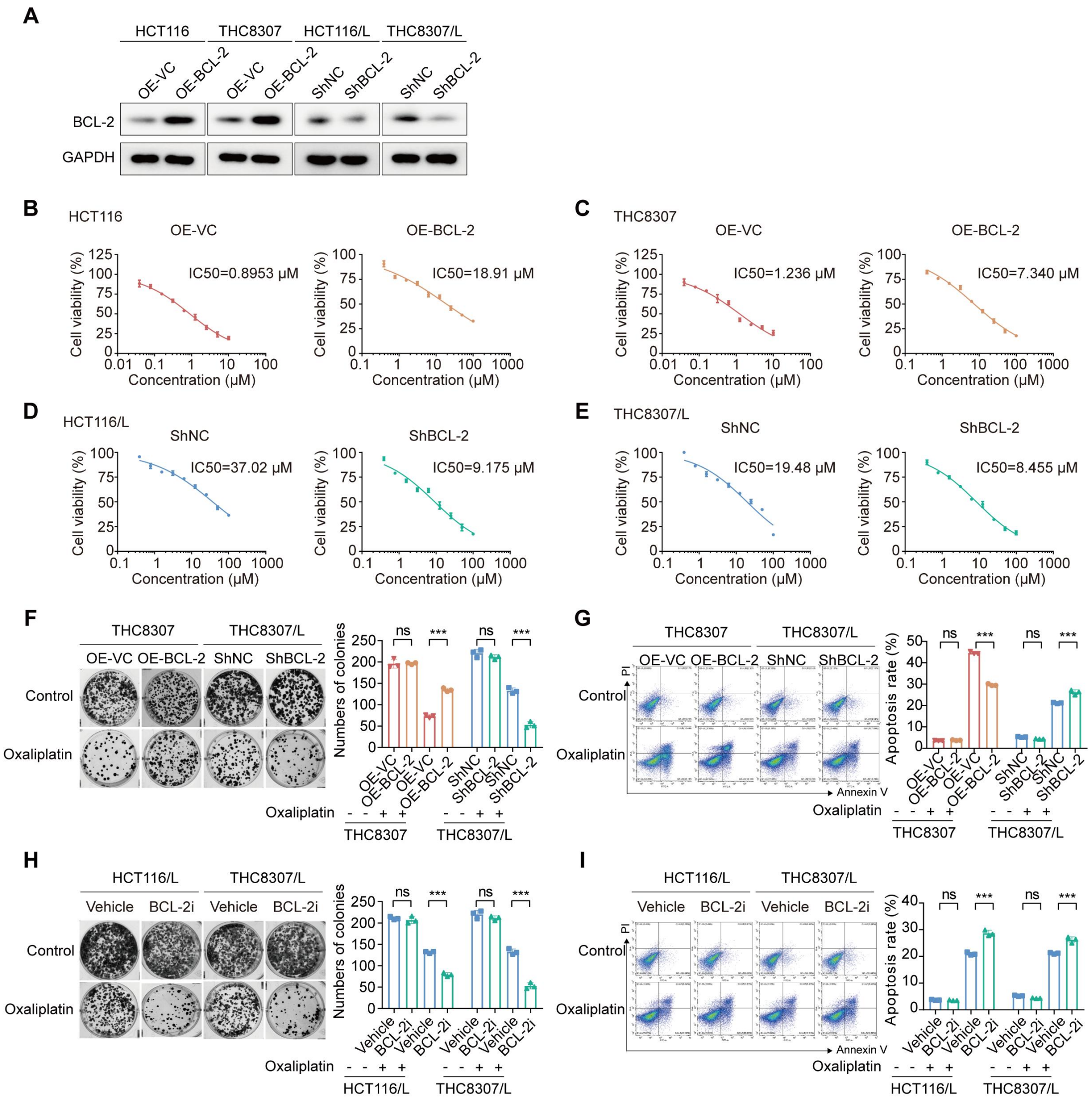

A. Western blot analysis showing the efficiency of BCL-2 overexpression and knockdown in HCT116, THC8307, HCT116/L, and THC8307/L cells.

B–E. IC_{50} values for oxaliplatin in HCT116 (B), THC8307 (C), HCT116/L (D), and THC8307/L (E) cells with BCL-2 overexpression or knockdown.

F. Representative images and quantification of colony formation in THC8307-OE-VC, THC8307-OE-BCL-2, THC8307/L-shNC, and THC8307/L-shBCL-2 cells treated with control or oxaliplatin (n = 3).

G. Representative images and quantification of Annexin V/propidium iodide staining in THC8307-OE-VC, THC8307-OE-BCL-2, THC8307/L-shNC, and

THC8307/L-shBCL-2 cells treated with control or oxaliplatin (n = 3).

H. Representative images and quantification of colony formation in HCT116/L and THC8307/L cells treated with vehicle or the BCL-2 inhibitor APG-2575, with or without oxaliplatin (n = 3).

I. Representative images and quantification of Annexin V/propidium iodide staining in HCT116/L and THC8307/L cells treated with vehicle or APG-2575, with or without oxaliplatin (n = 3).

Figure S3. A20 inhibits BCL-2 expression via the IKK-β/NF-κB/BCL-2 axis

Figure S3

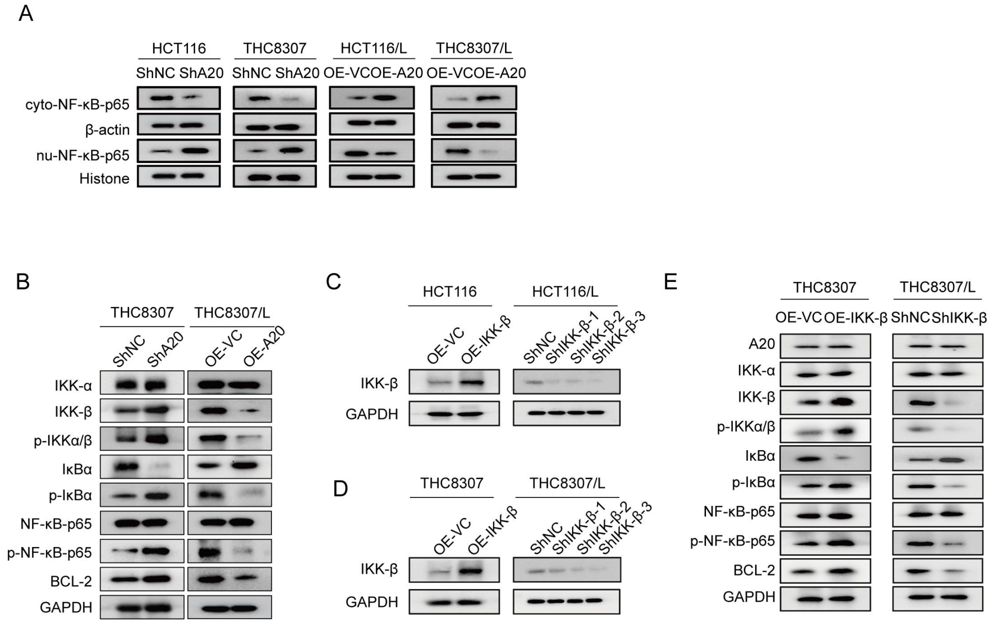

A. Cytoplasmic (cyto) and nuclear (nu) protein fractions from HCT116 and THC8307 cells with stable A20 knockdown, as well as HCT116/L and THC8307/L cells with A20 overexpression, were analyzed by Western blot for NF-κB-p65 expression.

B. Western blot analysis of IKK-α, IKK-β, p-IKKα/β, IκBα, p-IκBα, NF-κB-p65, p-NF-κB-p65, and BCL-2 expression in THC8307 cells with stable A20 knockdown and THC8307/L cells with A20 overexpression.

C–D. Western blot analysis showing the efficiency of IKK-β overexpression or knockdown in HCT116, HCT116/L, THC8307, and THC8307/L cells.

E. Western blot analysis of A20, IKK-α, IKK-β, p-IKKα/β, IκBα, p-IκBα, NF-κB-p65, p-NF-κB-p65, and BCL-2 expression in THC8307-OE-VC, THC8307-OE-IKK-β, THC8307/L-shNC, and THC8307/L-shIKK-β cells.

Figure S4. IKK-β expression regulates oxaliplatin resistance *in vitro*

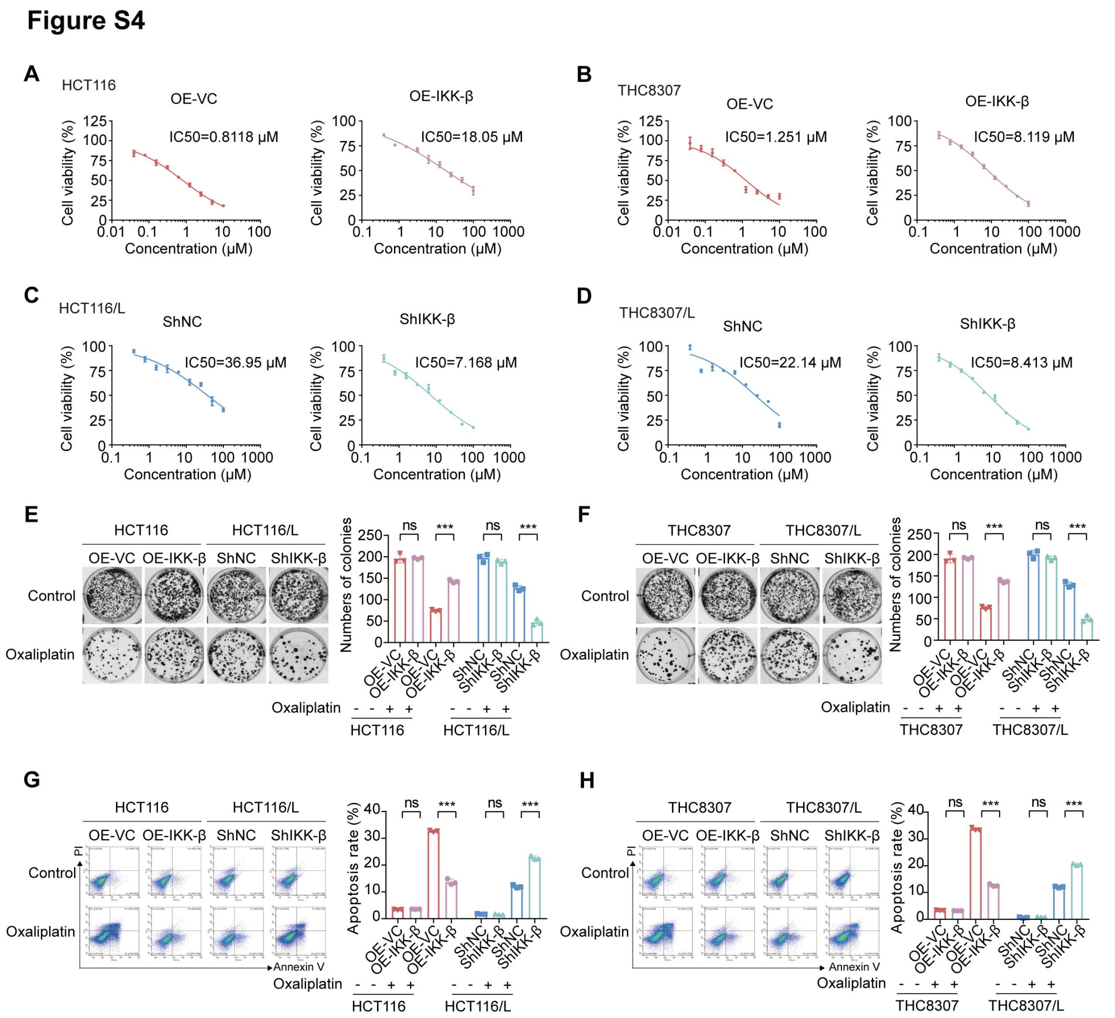

A–D. IC_{50} values for oxaliplatin in HCT116 (A), THC8307 (B), HCT116/L (C), and THC8307/L (D) cells with IKK-β overexpression or knockdown.

E. Representative images and quantification of colony formation in HCT116-OE-VC, HCT116-OE-IKK-β, HCT116/L-shNC, and HCT116/L-shIKK-β cells treated with control or oxaliplatin (n = 3).

F. Representative images and quantification of colony formation in THC8307-OE-VC, THC8307-OE-IKK-β, THC8307/L-shNC, and THC8307/L-shIKK-β cells treated with control or oxaliplatin (n = 3).

G. Representative images and quantification of annexin V/propidium iodide staining in HCT116-OE-VC, HCT116-OE-IKK-β, HCT116/L-shNC, and HCT116/L-shIKK-β cells treated with control or oxaliplatin (n = 3).

H. Representative images and quantification of annexin V/propidium iodide staining in THC8307-OE-VC, THC8307-OE-IKK-β, THC8307/L-shNC, and THC8307/L-shIKK-β cells treated with control or oxaliplatin (n = 3).

Figure S5. IKK-β and NF-κB inhibitors reverse oxaliplatin resistance induced by A20 deficiency in CRC cells

Figure S5

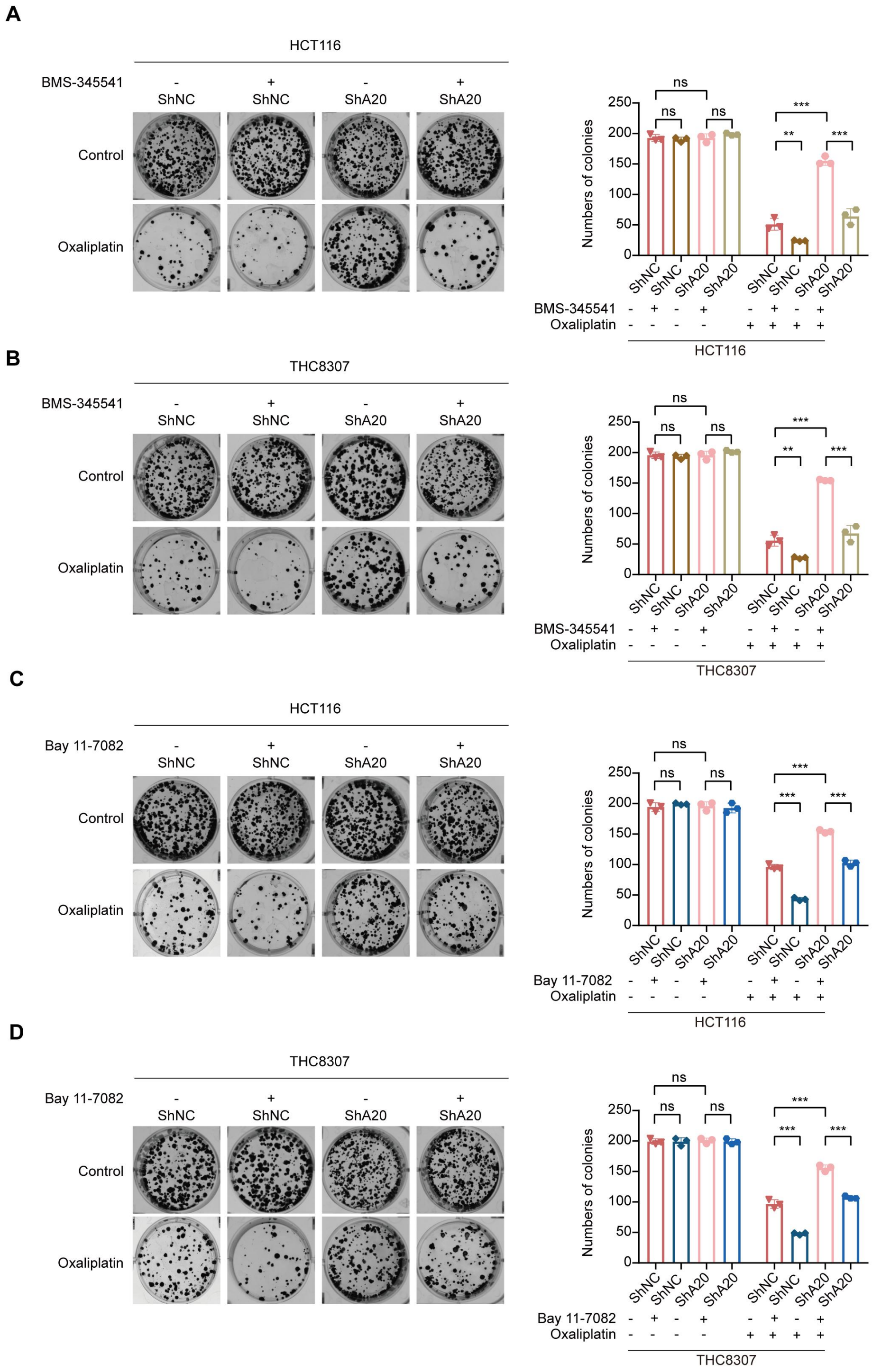

A. Representative images and quantification of colony formation in HCT116-shNC and HCT116-shA20 cells treated with control, oxaliplatin, or the IKK-β inhibitor BMS-345541 (n = 3).

B. Representative images and quantification of colony formation in THC8307-shNC and THC8307-shA20 cells treated with control, oxaliplatin, or BMS-345541 (n = 3).

C. Representative images and quantification of colony formation in HCT116-shNC and HCT116-shA20 cells treated with control, oxaliplatin, or the NF-κB inhibitor Bay 11-7082 (n = 3).

D. Representative images and quantification of colony formation in THC8307-shNC and THC8307-shA20 cells treated with control, oxaliplatin, or Bay 11-7082 (n = 3).

Figure S6. IKK-β expression regulates oxaliplatin resistance *in vivo*

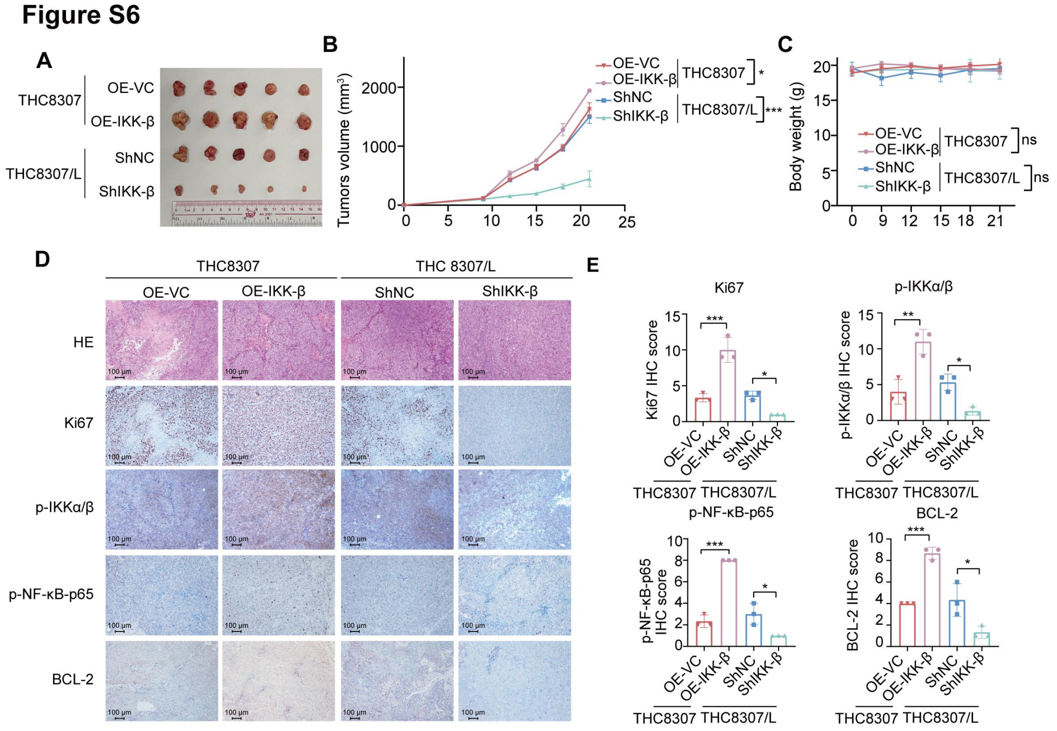

A. Representative images of tumors from BALB/c nude mice treated with oxaliplatin in the THC8307-OE-VC, THC8307-OE-IKK-β, THC8307/L-shNC, and THC8307/L-shIKK-β groups (n = 5).

B–C. Tumor growth curves (B) and body weight changes (C) of mice treated with oxaliplatin in the THC8307-OE-VC, THC8307-OE-IKK-β, THC8307/L-shNC, and THC8307/L-shIKK-β groups (n = 5).

D. Representative H&E staining and immunohistochemical staining of Ki67, p-IKKα/β, p-NF-κB-p65, and BCL-2 in tumor tissues from mice in the THC8307-OE-VC, THC8307-OE-IKK-β, THC8307/L-shNC, and THC8307/L-shIKK-β groups.

E. Quantification of Ki67, p-IKKα/β, p-NF-κB-p65, and BCL-2 immunohistochemical staining in tumors from mice in the THC8307-OE-VC, THC8307-OE-IKK-β, THC8307/L-shNC, and THC8307/L-shIKK-β groups (n = 3).

Figure S7. IKK-β protein levels are negatively correlated with A20

Figure S7

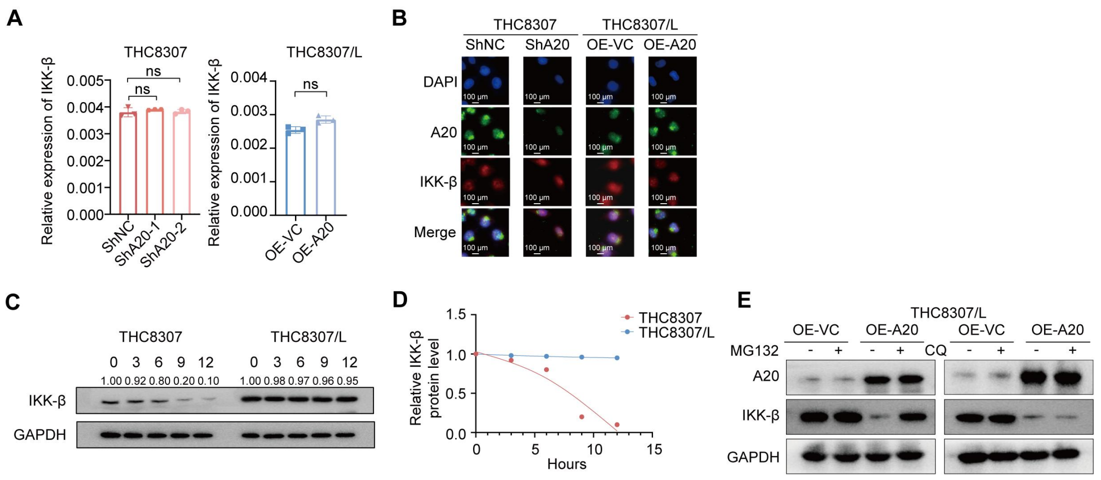

A. mRNA expression levels of IKK-β in THC8307 cells with stable A20 knockdown and in THC8307/L cells with A20 overexpression.

B. Representative immunofluorescence staining of DAPI, A20, and IKK-β in THC8307-shNC, THC8307-shA20, THC8307/L-OE-VC, and THC8307/L-OE-A20 cells.

C–D. Western blot analysis (C) and quantification (D) of IKK-β protein levels in THC8307 and THC8307/L cells treated with CHX (50 μM) for the indicated time points.

E. Western blot analysis of A20 and IKK-β expression in THC8307 cells with or without A20 overexpression, treated with or without MG132 (20 μM) or chloroquine (CQ, 40 μM).

Figure S8. The monoubiquitylated IKK-β lysine residue (K163) promotes oxaliplatin resistance *in vitro*

Figure S8

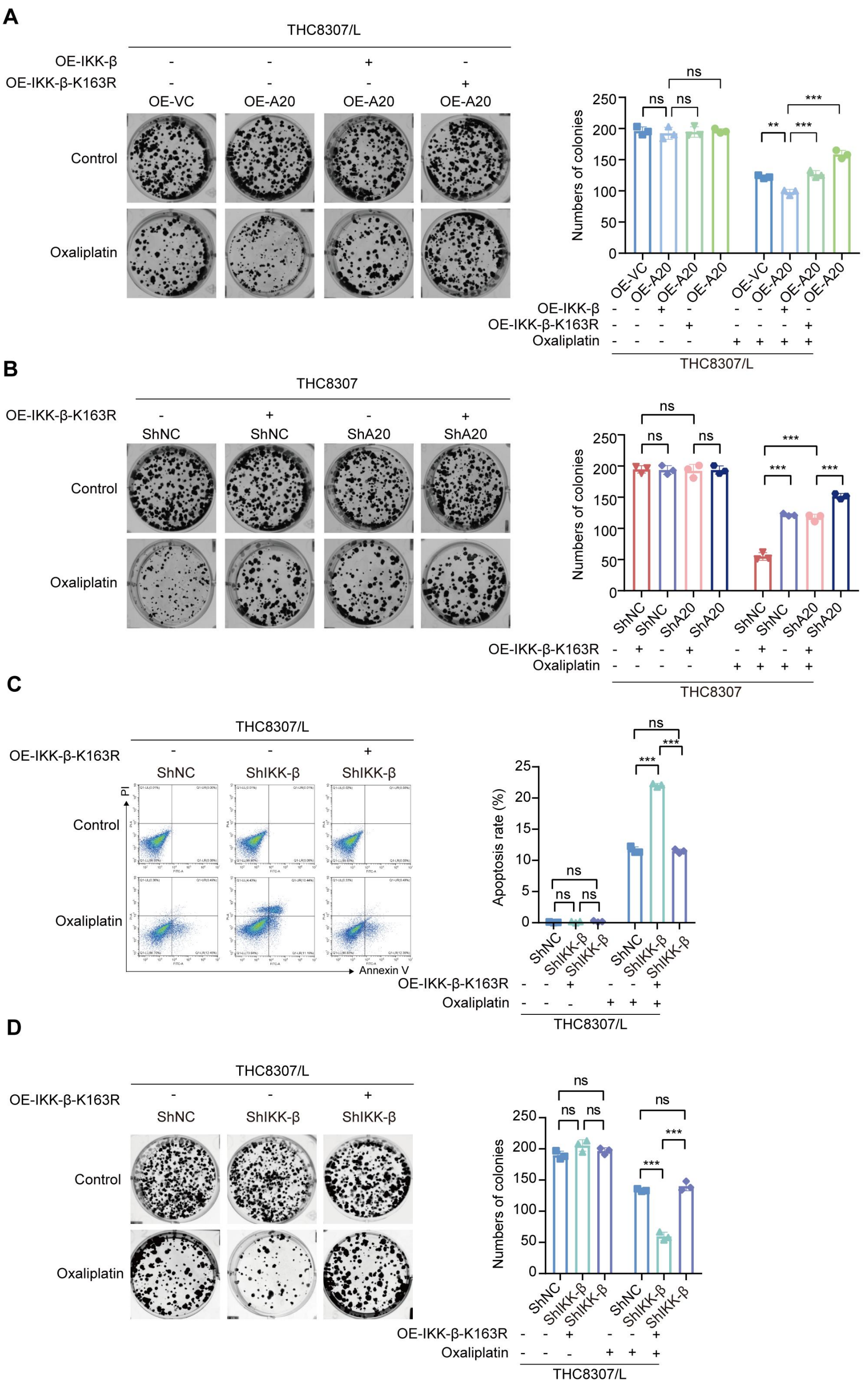

A. Representative images and quantification of colony formation in THC8307/L-OE-VC and THC8307/L-OE-A20 cells with or without wild-type or K163R mutant IKK-β overexpression, treated with control or oxaliplatin (n = 3).

B. Representative images and quantification of colony formation in THC8307-shNC and THC8307-shA20 cells with or without K163R mutant IKK-β overexpression, treated with control or oxaliplatin (n = 3).

C. Representative images and quantification of annexin V/propidium iodide staining in THC8307/L-shNC and THC8307/L-shIKK-β cells with or without K163R mutant IKK-β overexpression, treated with control or oxaliplatin (n = 3).

D. Representative images and quantification of colony formation in THC8307/L-shNC and THC8307/L-shIKK-β cells with or without K163R mutant IKK-β overexpression, treated with control or oxaliplatin (n = 3).

Supplementary Table 1. Characteristics of patients in cohort I (n = 85)

	Experimental group (n = 43)		Control group (n = 42)	
**Characteristics**	**Cases (n)**	**Percentage (%)**	**Cases (n)**	**Percentage (%)**
**Age (years)**				
Median (range)	63 (29-73)		58.5 (29-73)	
＜60	18	41.9	25	59.5
≥60	25	58.1	17	40.5
**Gender**				
Male	26	60.5	25	59.5
Female	17	39.5	17	40.5
**ypT-stage**				
T0	14	32.6	11	26.2
T1	1	2.3	2	4.8
T2	5	11.6	18	42.9
T3	17	39.5	6	14.3
T4	6	14	5	11.9
**N-stage**				
N0	37	86	35	83.3
N1	5	11.6	6	14.3
N2	1	2.3	1	2.4
**M-stage**				
M0	42	97.7	41	97.6
M1	1	2.3	1	2.4
**Disease stage**				
0	20	46.5	11	26.2
I	6	14	18	42.9
II	16	37.2	6	14.3
III	0	0	6	14.3
IV	1	2.3	1	2.4
**Neoadjuvant chemoradiotherapy**				
No	0	0	0	0
Yes	43	100	42	100
**Tumor Regression Grade**				
0	14	32.6	11	26.2
1	6	14	7	16.7
2	21	48.8	22	52.4
3	2	4.7	2	4.8
**pCR**				
No	14	32.6	11	26.2
Yes	29	67.4	31	73.8

Supplementary Table 2. Characteristics of all patients in cohort II.

Characteristics	Cases (n)	Percentage (%)
**Age (years)**		
Median (range)	56 (28-81)	
＜60	60	60
≥60	40	40
**Gender**		
Male	58	58
Female	42	42
**Histological score**		
1	4	4
2	9	9
3	81	81
4	2	2
5	1	1
6	3	3
**T-stage**		
T1	56	56
T2	37	37
T3	3	3
T4	4	4
**N-stage**		
N0	49	49
N1	18	18
N2	33	33
**M-stage**		
M0	89	89
M1	11	11
**Disease stage**		
I	43	43
II	1	1
III	45	45
IV	11	11
**Radiotherapy**		
No	81	81
Yes	19	19
**Extracranial metastatic site**		
Lung	9	9
Liver	4	4
Bone	3	3
Others	8	8
No	89	89
**DFS (months)**		
Median (range)	99.4 (9.6-136.1)	
**OS (months)**		
Median (range)	100.7 (14-136.1)	

Supplementary Table 3. Cox regression analysis of DFS in patients in the oxaliplatin group (cohort II).

	Univariate			Multivariate		
**Variables**	**OR**	**95% CI**	***p* Value**	**OR**	**95% CI**	***p* Value**
**Age** (<60 y vs. ≥60 y)	1.115	0.743-1.672	0.599			
**Gender** (female vs. male)	0.822	0.552-1.225	0.822			
**Pathological type** (1–3 vs. 5-6)	0.686	0.298-1.580	0.375			
**T-stage** (1-2 vs. 3-4)	1.428	0.688-2.963	0.339			
**N-stage** (0 vs. 1-2)	0.923	0.618-1.378	0.694			
**M-stage** (0 vs. 1)	0.535	0.074-3.891	**0.537**	2.238	0.786-6.373	0.131
**Disease stage** (I–II vs. III–IV)	0.946	0.630-1.421	0.789			
**Radiotherapy** (No vs. Yes)	0.839	0.507-1.388	**0.494**	2.282	0.970-5.367	0.059
**Extracranial metastatic site** (None vs. Exist)	0.421	0.239-0.739	**0.003**	0.271	0.105-0.695	**0.007**
**TNFAIP3** (Low vs. High)	1.213	0.875-1.567	**0.017**	2.312	0.923-4.567	**0.003**

Supplementary Table 4. Cox regression analysis of OS in patients in the oxaliplatin group (cohort II).

	Univariate			Multivariate		
**Variables**	**OR**	**95% CI**	***p* Value**	**OR**	**95% CI**	***p* Value**
**Age** (<60 y vs. ≥60 y)	1.435	0.522-3.945	**0.484**	0.604	0.214-1.703	0.131
**Gender** (female vs. male)	1.757	0.805-3.833	0.157			
**Pathological type** (1–3 vs. 5-6)	0.630	0.266-1.492	0.294			
**T-stage** (1-2 vs. 3-4)	0.632	0.282-1.413	0.263			
**N-stage** (0 vs. 1-2)	2.123	0.837-5.384	**0.113**	2.238	0.786-6.373	0.131
**M-stage** (0 vs. 1)	2.123	0.837-5.384	**0.113**	2.238	0.786-6.373	0.131
**Disease stage** (I–II vs. III–IV)	0.743	0.300-1.843	0.522			
**Radiotherapy** (No vs. Yes)	2.535	1.157-5.551	**0.020**	2.282	0.970-5.367	0.059
**Extracranial metastatic site** (None vs. Exist)	2.400	0.684-8.418	**0.113**	0.271	0.105-0.695	**0.007**
**TNFAIP3** (Low vs. High)	1.234	0.587-2.123	**0.0079**	1.799	0.845-4.213	**0.005**

Supplementary Table 5. Reagents used in this study.

REAGENT or RESOURCE	IDENTIFIER	SOURCE
Oxaliplatin	Cat# S1224	Selleck
Cell Counting Kit-8 (CCK-8) assay	Cat# CK04	Dojindo Laboratories
Annexin V-FITC cell apoptosis	Cat# C1062	Beyotime Biotechnology
Dual Luciferase Reporter Assay	Cat# E1910	Promega
Cycloheximide	Cat# S7418	Selleck
MG132	Cat# S2619	Selleck
Chloroquine	Cat# S6999	Selleck
Z-VAD-FMK	Cat# S7023	Selleck
Necrostatin-1	Cat# S8037	Selleck
Ferrostatin-1	Cat# S7243	Selleck
Disulfiram	Cat# S1680	Selleck
APG-2575	Cat# S9970	Selleck
BMS-345541	Cat# S8044	Selleck
Bay 11-7082	Cat# S2913	Selleck
**Reagents used in western blot and Co-IP Assay**		
BCL2 (RRID:AB_1903907)	Cat# 3498	Cell Signaling Technology
p-NF-κB-p65 (RRID:AB_331284)	Cat# 3033	Cell Signaling Technology
NF-κB-p65 (RRID:AB_10859369)	Cat# 8242	Cell Signaling Technology
p-IKKα/β (RRID:AB_2079382)	Cat# 2697	Cell Signaling Technology
IKK-α (RRID:AB_331626)	Cat# 2682	Cell Signaling Technology
IKK-β (RRID:AB_11024092)	Cat# 8943	Cell Signaling Technology
IKK-γ (RRID:AB_2124829)	Cat# 2685	Cell Signaling Technology
IKK-ε (RRID:AB_915926)	Cat# 2690	Cell Signaling Technology
p-IKBα (RRID:AB_561111)	Cat# 2859	Cell Signaling Technology
IKBα (RRID:AB_2799606)	Cat# 61294	Cell Signaling Technology
β-actin (RRID:AB_330288)	Cat# 4967	Cell Signaling Technology
Histone (RRID:AB_10544537)	Cat# 4499	Cell Signaling Technology
GAPDH (RRID:AB_561053)	Cat# 2118	Cell Signaling Technology
HA (RRID:AB_1549585)	Cat# 3724	Cell Signaling Technology
Flag (RRID:AB_2572291)	Cat# 14793	Cell Signaling Technology
A20 (RRID:AB_10698880)	Cat# 5630	Cell Signaling Technology
XIAP (RRID:AB_2214870)	Cat# 2042	Cell Signaling Technology
BAKM (RRID:AB_2716685)	Cat# 12105	Cell Signaling Technology
BCL-XL (RRID:AB_2228008)	Cat# 2764	Cell Signaling Technology
BAD (RRID:AB_2062127)	Cat# 9239	Cell Signaling Technology
Ubiquitin (RRID:AB_2799235)	Cat# 43124	Cell Signaling Technology
Rabbit IgG (RRID:AB_1031062)	Cat# 2729	Cell Signaling Technology

Protein A/G Magnetic Beads	Cat# 88802	Thermo Fisher
**Reagents and Recombinant DNA used in Cell transfection**		
Lipofectamine 3000 transfection reagent	Cat# L3000015	Life Technologies
pGL3-basic	Cat# E1751	Promega
pGL3-basic-BCL-2 promoter	Cat# G46906	MiaoLing
pCDNA3.1-BCL-2	Cat# P8816	MiaoLing
pCMV-3×MYC-RELA (human)-Neo	Cat# P60487	MiaoLing
pCDNA3.1-Ubiquitin-WT	Cat# GNS-1013	GrowGn
pCDNA3.1-Ubiquitin-7KR	Cat# GNS-1014	GrowGn
pCDNA3.1-6×His-Ubiquitin-WT	Cat# P28355	MiaoLing
pCDNA3.1-6×His-Ubiquitin-K48R	Cat# P7778	MiaoLing
pCDNA3.1-6×His-Ubiquitin-K63R	Cat# P7779	MiaoLing
pCDNA3.1-6×His-Ubiquitin-K6	Cat# G43227	MiaoLing
pCDNA3.1-6×His-Ubiquitin-K11	Cat# G43228	MiaoLing
pCDNA3.1-6×His-Ubiquitin-K27	Cat# G43229	MiaoLing
pCDNA3.1-6×His-Ubiquitin-K29	Cat# G43230	MiaoLing
pCDNA3.1-6×His-Ubiquitin-K33	Cat# G43231	MiaoLing
pCDNA3.1-6×His-Ubiquitin-M1R	Cat# G47368	MiaoLing
pReceiver-M07-3HA-NC	Cat# EX-NEG-M07	GeneCopoia
pReceiver-M07-3HA-IKKβ-WT	Cat# EX-K6125-M07	GeneCopoia
pCDNA3.1-3×HA-C	Cat# P6808	MiaoLing
pCDNA3.1-IKKβ-3×HA	Cat# P10234	MiaoLing
pCDNA3.1-IKKβ-K163R-3×HA	Cat# G39534	MiaoLing
pCDNA3.1-IKKβ-S177E-3×HA	Cat# G43289	MiaoLing
pCDNA3.1-IKKβ-C179E-3×HA	Cat# G43287	MiaoLing
pCDNA3.1-IKKβ-S181E-3×HA	Cat# G43288	MiaoLing
pReceiver-M12-3×Flag-NC	Cat# EX-NEG-M12	GeneCopoia
pReceiver-M12-3×Flag-A20-WT	Cat# EX-K6040-M12	GeneCopoia
pReceiver-M12-3×Flag-A20-ΔZNF4	Cat# EX-K6040-M12-02	GeneCopoia
pReceiver-M12-3×Flag-A20-ΔZNF7	Cat# EX-K6040-M12-09	GeneCopoia
pCMV-3×Flag-N	Cat# P0860	MiaoLing
pCMV-3×Flag-A20	Cat# P0860-001	MiaoLing
pCMV-3×Flag-A20-Y614A/F615A	Cat# G9674	MiaoLing
pCMV-3×Flag-A20-C624A/C627A	Cat# G9673	MiaoLing
pCMV-3×Flag-A20-F770A/G771A	Cat# G9675	MiaoLing

Lentiviral particles for Scrambled Control	GXDL0241833	Shanghai Genechem
Lentiviral particles for BCL-2	GXDL0241833	Shanghai Genechem
Lentiviral particles for IKBKB-wild type	LPP-NEG-Lv201-A00	GeneCopoeia Inc.
Lentiviral particles for IKBKB-K163R	LPP-CS-K6125-Lv201-03-A00	GeneCopoeia Inc.
Lentiviral particles for shNC	LV-BCL2-RNAi(15921-1)	Shanghai Genechem
Lentiviral particles for shBCL-2	LV-BCL2-RNAi(15921-1)	Shanghai Genechem Co.,Ltd.
Lentiviral particles for shIKBKB#1	HSH054492-LVRU6GP-a	GeneCopoeia Inc.
Lentiviral particles for shIKBKB#2	HSH054492-LVRU6GP-b	GeneCopoeia Inc.
Lentiviral particles for shIKBKB#3	HSH054492-LVRU6GP-c	GeneCopoeia Inc.
**Reagents used in qRT-PCR**		
TRIzol™ Reagent	Cat# 10296010	Invitrogen
Reverse transcription supermix kit	Cat# 1708840	Bio-Rad
Reverse transcription supermix kit	Cat# 1708840	Bio-Rad
**Reagents used in IHC**		
Ki67 (RRID:AB_2636984)	Cat# 9027	Cell Signaling Technology
Bcl-2 (RRID:AB_2715467)	Cat# ab182858	Abcam
A20 (RRID:AB_2882048)	Cat# 66695-1-Ig	Proteintech
p-NF-κB-p65 (RRID:AB_1925243)	Cat# ab86299	Abcam
p-IKKα/β (RRID:AB_2079382)	Cat# 2697	Cell Signaling Technology
**Reagents used in Immunofluorescence Analysis**		
4 ',6-diamino-2-phenylindole	Cat# 2201593	Invitrogen
A20 (RRID:AB_2882048)	Cat# 66695-1-Ig	Proteintech
IKK-β (RRID:AB_3712116)	Cat# abs158775	Absin
Flag (RRID:AB_10950495)	Cat# 8146	Cell Signaling Technology
HA (RRID:AB_10691311)	Cat# 2367	Cell Signaling Technology
**Experimental models: Cell lines**		
HCT116	Cat# CCL-247	ATCC
THC8307	Cat# CL0854	Fenghui Biotechnology
HEK 293T	Cat# CRL-11268	ATCC
**Software and algorithms**		
FlowJo softw	N/A	BD
GraphPad Prism	N/A	GraphPad Software
SPSS Statistics 19 software	N/A	IBM

Supplementary Table 6. Sequences of primers for DNA constructs and oligonucleotides.

Sequence Name	Sequence
Primer sequences	
A20-F	GATAGAAATCCCCGTCCAAGG
A20-R	CTGCCATTTCTTGTACTCATGC
Oligonucleotide sequences	
ShA20#1	GGTTGTAGATGCTGGTCTTTG
ShA20#2	GGTGGAAACTTCTTTCCTTGT
ShA20#3	GCAGGAATCTCAGGTCCTTAT
ShA20#4	GCACTTGCCAAAGGAGATTAA
